# Daily sampling reveals rapid microbiota alterations and antimicrobial resistance gene acquisition during intercontinental travel

**DOI:** 10.1038/s41522-026-00977-x

**Published:** 2026-04-07

**Authors:** Jiyang Chan, Christian J. H. von Wintersdorff, Paul H. M. Savelkoul, Niels van Best, Petra F. G. Wolffs, John Penders

**Affiliations:** 1 Department of Medical Microbiology, Infectious diseases and Infection Prevention, Institute of Nutrition and Translational Research in Metabolism (NUTRIM), Maastricht University Medical Centre+, Maastricht, The Netherlands; 2 Department of Medical Microbiology, Infectious diseases and Infection Prevention, Institute for Public Health and Primary Care (Caphri), Maastricht University Medical Centre+, Maastricht, The Netherlands; 3 Institute of Medical Microbiology, RWTH Aachen University Hospital, RWTH Aachen University, Aachen, Germany

**Keywords:** Diseases, Microbiology

## Abstract

With increasing global travel, individuals are frequently exposed to new diets, environments, and microbial communities that may influence gut microbiota dynamics and facilitate the acquisition of antimicrobial resistance genes (ARGs). At present, studies characterising day-to-day microbiota and ARGs dynamics during travel are lacking. This study aimed to elucidate the short-term effects of travel on gut microbiota dynamics and ARG acquisition using a high-frequency sampling approach. A cohort of eleven Dutch travellers to Asia self-collected 254 fecal swabs before, during, and after travel for microbiota and resistome profiling. Samples were analysed using qPCR targeting clinically relevant ARGs (*qnrB, qnrS* and *bla*_CTX-M_) and profiled by 16S rRNA gene amplicon sequencing. Longitudinal analyses revealed pronounced inter- and intra-individual variation, with rapid shifts in microbiota composition observed within the first days of travel. An increase in Enterobacterales and a decline in commensal taxa were detected during early travel, coinciding with swift ARG acquisition. These findings underscore the key role of travel in global ARG dissemination.

## Introduction

In our modern world, the increase in accessibility of global travel offers opportunities for cultural exchange, business opportunities, and leisure activities. However, this heightened mobility also presents unique public health challenges, as increased mobility facilitates exposure to, and the transboundary spread of, geographically restricted pathogens as well as antimicrobial-resistant microbes. The most common health consequence of such exposures is the onset of gastrointestinal infections^1^.

Beyond acute infections, growing attention has been given to the role of the gut microbiota in maintaining gut health, and by extension, the overall well-being^2–4^. Disruptions in the gut microbiota can not only accompany symptoms but also serve as early indicators of susceptibility to various diseases^5–8^. A healthy adult gut microbiota is resilient to perturbations and stable over years, although slight day-to-day variations have been observed, and major dietary shifts and lifestyle changes have the potential to alter the microbiota^9–11^. Such shifts potentially readily happen during travel to foreign countries due to the exposure to unfamiliar cuisines, environments, microbial exposures, vaccinations, (prophylactic) medications or complications including traveller’s diarrhoea.

Several studies have examined the impact of international travel on the gut microbiota^12–18^. However, most of the comparisons in these studies were performed between pre-travel and post-travel observations, with only a couple of studies investigating the temporal microbiota variability during travel. A case study on an individual who alternated a two-month stay in Italy with a two-month stay in Nigeria demonstrated that short-term geographical changes could result in shifts in gut microbiota structure^12^. Boolchandani et al.^13^ observed within a cohort of 159 international students travelling to Peru that diarrhoea disrupted the stability of the taxonomic diversity. Nonetheless, the results of both studies were based on weekly sampling intervals, which may not adequately capture the rapid shifts and day-to-day variability of the gut microbiota.

Moreover, intercontinental travel also serves as a potential vector for the dissemination of antimicrobial resistance genes (ARG). Previous research has shown that human travel to areas with a high prevalence of antimicrobial resistance (AMR) contributes to the spread of AMR bacteria or ARGs across geographic areas^19^. Yet, there are currently no published studies that characterise day-to-day changes in the resistome in the context of travel. Here, we collected fecal swabs at high-resolution from 11 intercontinental travellers taken before, daily during, and at several time points after their journeys to investigate whether and how travel alters the composition of the gut microbiota. In addition, we screened fecal as well as skin microbiomes for several ARGs to assess the rate of ARG acquisition.

## Results

In total, we investigated 11 travelling individuals, 6 females and 5 males. Travellers (Tr) 01 and Tr04 are the same individual who underwent two separate trips in different years (Table 1). The median duration of travel was 14 (IQR 11–22) days. All travellers visited countries in Asia (Table 1). Furthermore, Tr01 and Tr02 were companions who travelled to the same country. Tr08, Tr09, Tr11 and Tr12, were a family of four who travelled together to Malaysia. None of the participants was hospitalised, and none reported antibiotic use during travel or within the three months preceding or following travel. Both Tr01 and Tr11 suffered from a diarrhoeal episode during their trip.

**Table 1:** Characteristics of participants

Traveller (Tr) #	Sex	Age	Destination	Duration (days)	Diarrhoea during travel
01	M	24	South Korea	12	Yes, day 10
02	F	24	South Korea	12	No
03	M	37	India	21	No
04	M	24	India	10	No
05	F	38	China	21	No
07	M	25	Philippines	14	No
08	F	7	Malaysia	14	No
09	F	10	Malaysia	23	No
11	M	40	Malaysia	23	Yes, days 15 & 16
12	F	38	Malaysia	23	No
13	M	51	India	5	No
14	F	42	India	5	No

### Transient and individual-specific changes in gut microbial diversity during and after travel

**Fig. 1:**
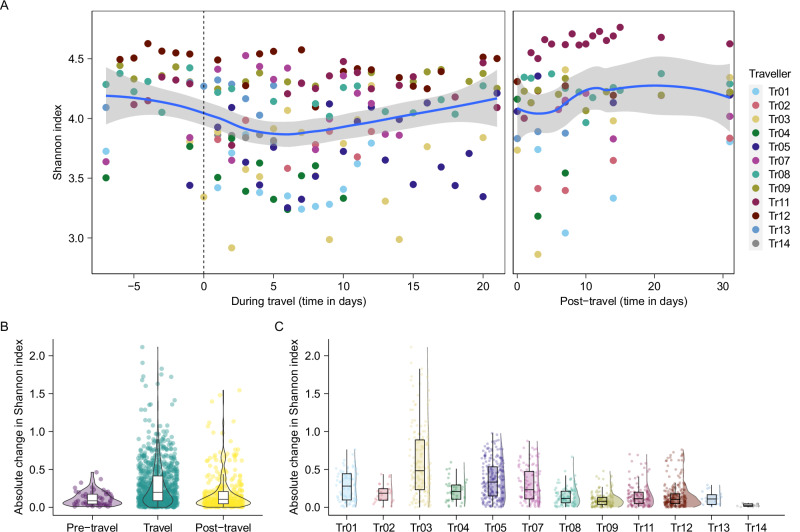
Temporal dynamics of gut microbiota diversity in travellers. **A** Shannon diversity over time per traveller. Blue line depicts fitted Locally Estimated Scatterplot Smoothing (LOESS) curve. The standard error (SE) confidence band is displayed in grey. Dashed line depicts the day of departure. **B** Intra-individual absolute changes in Shannon diversity, showing pairwise comparisons between samples within each individual per period. **C** Inter-individual absolute changes in Shannon diversity. Box plots denote the median, IQR, and 95% quantiles.

Altogether, 254 fecal swabs (33 pre-travel, 142 during travel and 79 post-travel) from twelve travel episodes involving eleven individual participants were subjected to DNA extraction, 16S rRNA gene amplicon sequencing, qPCR targeting ARGs, and downstream bioinformatics data pre-processing to enable analysis in gut microbiota dynamics and ARGs in travellers. The microbial diversity (Shannon) of the travellers’ gut microbiota trended towards a decline directly after arrival to the index country (Fig. 1A). Subsequently, several travellers showed decreased microbial diversity within 5 days after travel compared to their last sample collected during travel (Fig. 1A). Despite these apparent declines in diversity after arrival at the travel destination and again upon travel return, generalised linear mixed-effects models showed no statistically significant differences between pre-travel, during travel, and post-travel time periods (all Tukey-adjusted *P* > 0.2; Fig. 1B). This suggests that there are no consistent shifts in microbial diversity on a population level. Indeed, considerable inter-individual variability in changes in Shannon diversity was observed (Fig. 1C), suggesting host-specific microbiota dynamics in response to exposures within the different time periods.

### Travel induces temporary reductions in gut microbiota stability amid individual signatures

**Fig. 2:**
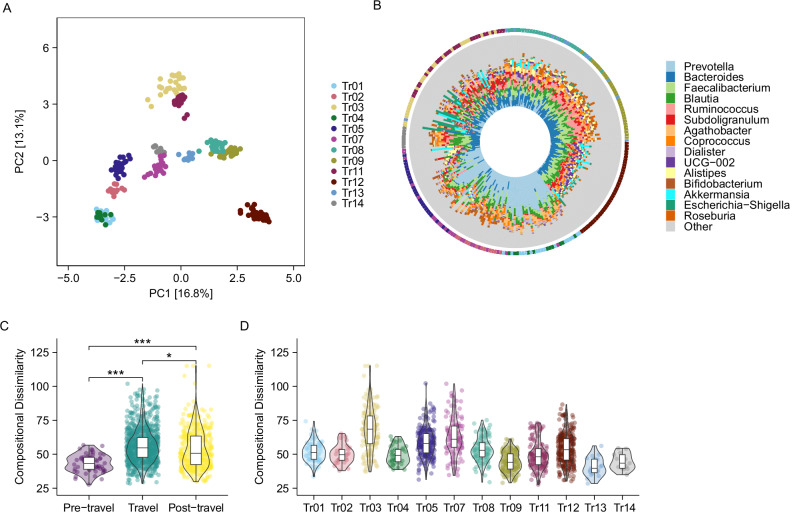
Temporal and inter-individual variation in gut microbiota composition in travellers. **A** PCA visualising beta-diversity of the traveller cohort across all time periods (pre-, during-, and post-travel), with each traveller represented by a unique colour. **B** Circular bar plot arranged by PCA sample positions, with an outer ring indicating individual travellers using the same colour scheme. **C** Intra-individual compositional dissimilarity per period, shown as pairwise Aitchison distances between samples within each individual per period (linear mixed-effects modelling and post hoc estimated marginal means analysis and corrected for multiple testing with Tukey’s HSD, ***P < 0.001, *P < 0.05). **D** Inter-individual compositional relatedness of travellers based on pairwise Aitchison distances. Box plots denote the median, IQR, and 95% quantiles.

Next, we investigated the microbial community composition. Principal Component Analysis (PCA) demonstrated clear inter-individual differences in composition, with each individual maintaining a unique microbiota profile over time (Fig. 2A, B). Notable exceptions are Tr01 and Tr04, as these observations come from the same person and appear to cluster together in the PCA plot (Fig. 2A). Intra-individual variation in microbiota composition differed significantly across time periods, with the highest day-to-day dissimilarity observed during travel, indicating reduced microbial stability compared to pre- and post-travel (Tukey-adjusted *P* < 0.001; Fig. 2C).

Consistent with the inter-individual differences in microbial diversity dynamics, we also observed substantial variation in compositional stability across travellers, with some showing highly stable microbiota and others exhibiting pronounced temporal fluctuations (Fig. 2D).

**Fig. 3:**
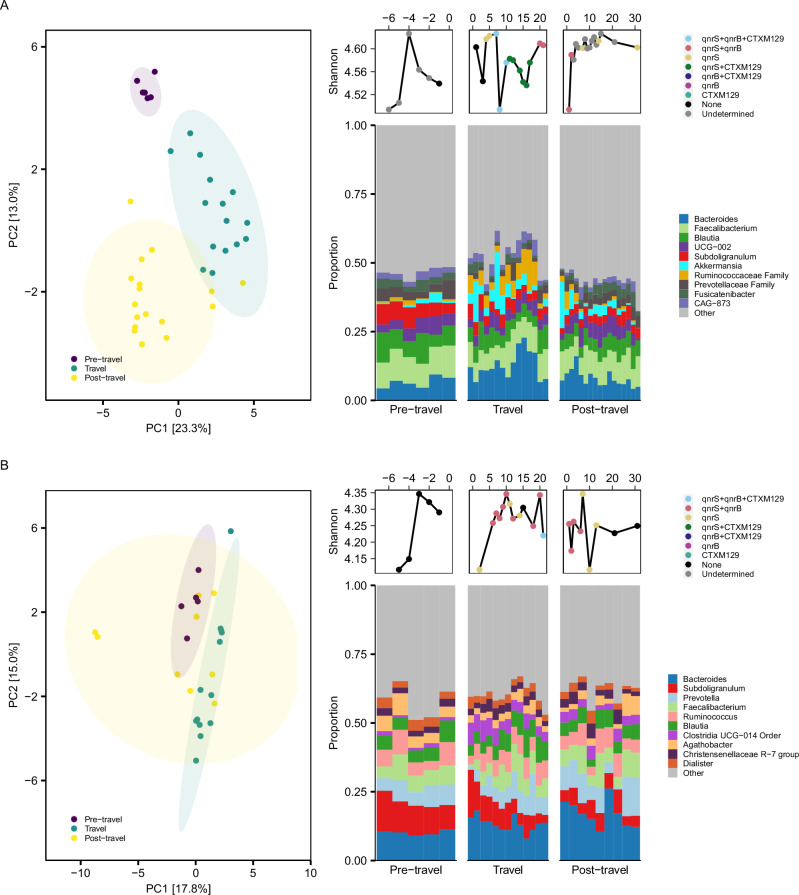
Longitudinal gut microbiota composition and diversity in representative travellers. **A** Microbial composition and diversity in traveller subjects over time of Tr12 (top plots) and **B** Tr11 (bottom plots). Principal component analysis (PCA) of the gut microbiota coloured by time period (pre-, during-, and post-travel). Shannon diversity is plotted over time, with collection points coloured by the presence of antimicrobial resistance genes (ARGs) detected at each time point. Bar plots show the relative abundance of the top 10 bacterial genera per time period, with bars positioned chronologically by sampling time points within each period.

To further explore the differences in microbial community structure between each time period (before-, during- and after travel), we visualised the dynamics in microbial (Shannon) diversity and composition for each of the travellers separately (Fig. 3, Supplementary Figs. 1–10). Day to day variation in Shannon diversity and bacterial community structure was observed with differences between individuals, but also between time periods within the same individuals.

As an example, distinct clustering of samples per period was observed in the PCA of Tr12 (Fig. 3A). The Euclidean distance between subsequent samples of this female traveller prior to travel was lower compared to the distances observed between subsequent samples within the other time periods (during travel and post-travel), indicating a more stable microbiota profile prior to travel. Longitudinal changes in Shannon diversity were assessed relative to the individual pre-travel baseline diversity (the sample closest to travel departure). These descriptive analyses revealed that the microbial diversity remained relatively stable during the pre-travel period, i.e. values remaining close to the individual baseline (Supplementary Fig. 11A). During travel, diversity showed pronounced temporal variability, characterised by several transient decreases ranging approximately from −0.05 to −0.44 relative to baseline, followed by partial recovery toward baseline levels. Immediately after travel, a marked decline in diversity was observed, reaching −0.54 relative to baseline. Subsequently, Shannon diversity gradually increased over time, with values approaching baseline levels and appearing to stabilise after 30 days post-travel. In addition, the relative abundances of *Bacteroides*, *Akkermansia*, and members of the Ruminococcaceae family exhibited transient fluctuations during travel, with increases relative to pre-travel baseline values and day-to-day variability, followed by a gradual return toward baseline levels after travel (Supplementary Fig. 12A). These temporal changes in microbial diversity and community composition suggest reduced stability of the gut microbiota during the travel period.

In another example, the male traveller Tr11, who accompanied Tr12 to Malaysia, had distinct clustering of pre-travel and travel samples while the post-travel samples clustering partly overlapped with the others and showed highest temporal instability (Fig. 3B). Here, the shift of gut microbiota was characterised by fluctuations in *Subdoligranulum* and *Clostridia UCG-014* (Supplementary Fig. 12B). Although Tr11 suffered from diarrhoeal episodes on day 14 and day 15 of travel, no large reductions in Shannon diversity were observed except for the initial drop during the first day of arrival (Supplementary Fig. 11B). Noticeably, the offspring (Tr08 and Tr09, both females) of Tr11 and Tr12 also displayed different clustering between pre-travel, during travel and post-travel samples in their individual PCA’s (Supplementary Figs. 7 and 8). Their samples clustered between the samples of Tr11 and Tr12 (Fig. 2A), suggesting a partially shared microbiota between the children and their parents. Given that this family engaged in a round-trip backpacking journey and consumed local street food for most of the trip, representing a marked departure from their usual diet, findings suggest that abrupt dietary shifts may contribute to the pronounced microbiota changes observed.

### Rapid and frequent acquisition of ARGs in travellers

**Fig. 4:**
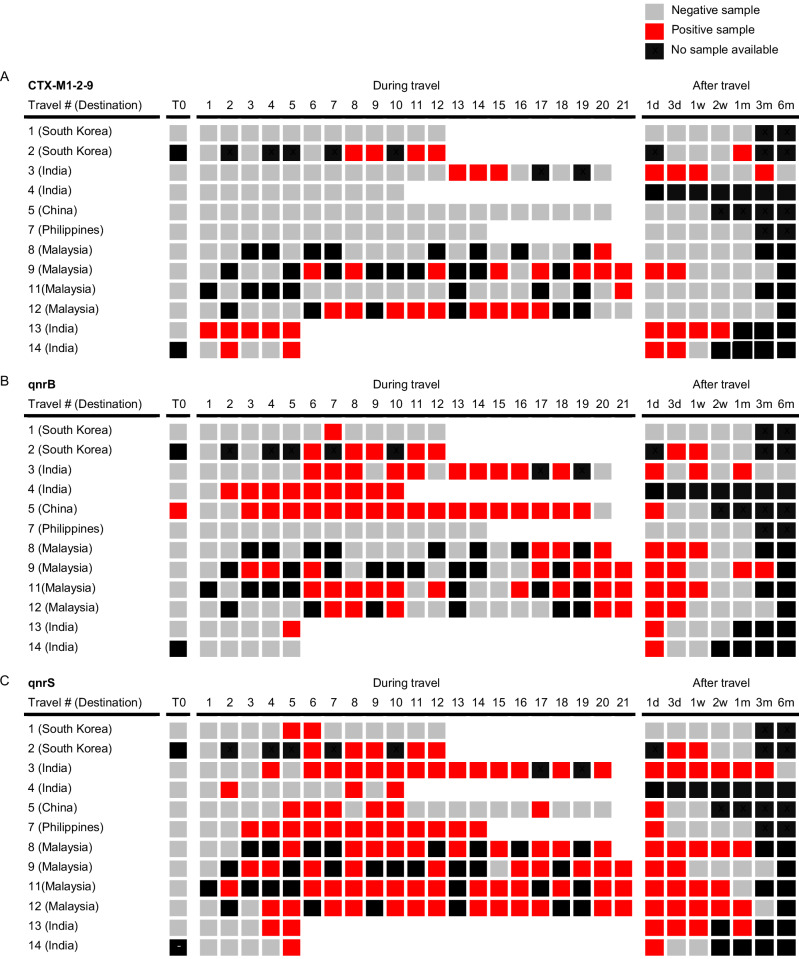
Temporal detection of antimicrobial resistance genes in travellers. Presence of **A** *bla*_CTX-M_, **B** *qnrB* and *qnrS* **C** genes in fecal swabs before, during and after international travel. Grey squares represent negative swabs, while red squares indicate positive samples. Black squares indicate time points at which no swab was collected. T0: 1 day before travel or day of departure; 1 d: 1 day; 3 d: 3 days; 1w: 1 week; 2w: 2 weeks; 1m: 1 month; 3m; 3 months; 6m: 6 months.

Thereafter, we performed qPCRs targeting ARGs encoding clinically relevant extended-spectrum beta-lactamases (*bla*_CTX-M_ groups 1, 2, and 9) and plasmid-mediated quinolone resistance (*qnrS*, *qnrB*). Analyses revealed that all the participants had newly acquired at least one of these AMR genes during travel, absent in their pre-travel fecal swabs (Fig. 4). Pre-travel ARG detection was nearly absent (1.67%) and entirely driven by a single participant with a *qnrB*-positive fecal swab collected four days prior to departure (Tr08; Supplementary Figs. 7 and 13). All other travellers were pre-travel negative (Supplementary Fig. 13), supporting sporadic low-abundance carriage near the analytical detection limit rather than established pre-travel colonisation. In contrast, detection rates increased during travel, most prominently in the first week, and remained detectable beyond travel in a subset of participants, consistent with travel-associated acquisition and, in some cases, persistent post-travel carriage (Fig. 4, Supplementary Fig. 13). Group-level analysis of traveller-level positivity (defined as the percentage of samples testing positive within each period) revealed significant differences across travel periods for *bla*_CTX-M_, *qnrB* and *qnrS* (Friedman test, *P* = 0.014, *P* = 0.001 and *P* = 0.007, respectively). Post hoc analyses revealed increased detection of *qnrB* and *qnrS* during travel compared with pre-travel (paired Wilcoxon signed-rank test, raw *P* = 0.008, adjusted *P* = 0.047 for both). A similar increase was observed for *bla*_CTX-M_ (raw *P* = 0.036), although this did not remain statistically significant after multiple-testing correction (adjusted *P* = 0.177). Together, these findings support travel-associated acquisition of AMR genes. Detection rates declined after 1 week post-travel for *bla*_CTX-M_ and *qnrB* compared to during travel (paired Wilcoxon signed-rank test, *bla*_CTX-M_ *P* = 0.059, adjusted *P* = 0.177; *qnrB* *P* = 0.036, adjusted *P* = 0.072), whereas no difference was observed for *qnrS* (*P* = 0.25, adjusted *P* = 0.30), suggesting more persistent carriage.

Across several travellers, we observed intermittently negative samples characterised by runs of consecutive ARG-positive time points interrupted by isolated negative measurements (Fig. 4). Such “on–off” patterns are likely more consistent with fluctuations in bacterial biomass and target abundance around the limit of detection than with true acquisition or complete loss events. While on average, the total bacterial load (16S rRNA gene copy numbers) did not differ markedly across detection patterns (e.g., intermittent non-detection versus sustained positivity, or pre-travel negative states, Supplementary Fig. 14), inspection of individual trajectories suggested that intermittent non-detection of AMR genes could, at least in part, be explained by low bacterial DNA load in swabs collected during travel. For example, in traveller Tr03, 16S rRNA gene quantification indicated reduced bacterial DNA content coinciding with intermittently negative time points, consistent with transient non-detection below the analytical threshold rather than genuine clearance (Supplementary Fig. 15).

To further evaluate specimen-related sensitivity, a subset of participants provided paired fecal stool samples alongside swabs (Supplementary Fig. 16). Overall concordance between stool and swab testing was 81% (51/63 paired analyses). Among discordant pairs, stool samples were more often positive while the paired swabs were negative (9/12 discordant pairs; 14% of all paired analyses), consistent with lower sensitivity of swab-based detection compared to stool specimens. The opposite pattern (swab positive, stool negative) was less common (3/12 discordant pairs; 5% of all paired analyses), suggesting that inhibition or technical interference in stool accounted for only a minority of discrepancies.

Collectively, the trajectory analyses and stool–swab comparisons support travel-associated acquisition and persistent carriage in a subset, while underscoring that swab-based testing can yield intermittent false negatives when bacterial DNA content is low.

The most frequently acquired ARGs were associated with quinolone resistance (Fig. 4B, C; Supplementary Fig. 16B, C). The *qnrB* gene was detected in fecal samples of 8 of 10 travellers who did not carry this gene prior to travel. Notably, intestinal acquisition of *qnrB* occurred as early as day 2 of travel for one participant (Tr04, India). The persistence of *qnrB* carriage varied widely between participants. In one traveller (Tr01, South Korea), *qnrB* was detected at only a single time point during travel, whereas in Tr09 *qnrB* remained detectable until the final sample collection at 3 months post-travel. In Tr03, hand skin swabs were found to be positive for *qnrB* on days 2 and 3 (Supplementary Fig. 17B), after which this gene was also recovered from the participant’s fecal swabs from travel day 6 onwards, suggesting fecal-oral transmission.

The *qnrS* gene, absent in all pre-travel fecal samples, was acquired by all travellers (Fig. 4C and Supplementary Fig. 16C). In 4 out of 9 travellers with follow-up samples at one month post-travel, *qnrS* was still detectable. Acquisition of *bla*_CTX-M_ genes was detected in 7 participants (Fig. 4A and Supplementary Fig. 16A), although Tr14 already had a positive pre-travel stool sample. The earliest acquisition was detected on day one of travel in Tr13 (India). Hand skin swabs collected during Tr04’s trip were positive for *bla*_CTX-M_ on days 2 and 6 (Supplementary Fig. 17A), while the fecal swabs collected during the same trip remained negative.

### Enterobacterales expansion and compositional shifts in commensal taxa accompany ARG acquisition during travel

We next investigated whether the gut microbiota was associated with the acquisition or presence of AMR genes. To examine whether a lower microbial diversity increased the risk of ARG acquisition during travel, we limited our analysis to *bla*_CTX-M_, as *qnrB* and *qnrS* were acquired by (almost) all participants. No significant association was found between Shannon index and *bla*_CTX-M_ acquisition (LMM; *P* = 0.08; Supplementary Fig. 18). Group-level analysis showed no significant associations across acquisition phases (before AMR gene acquisition; post-acquisition phase positive for the AMR gene; post-negative: post-acquisition phase negative for the AMR gene; paired Wilcoxon rank sum test *P* > 0.05; Supplementary Fig. 19).

**Fig. 5:**
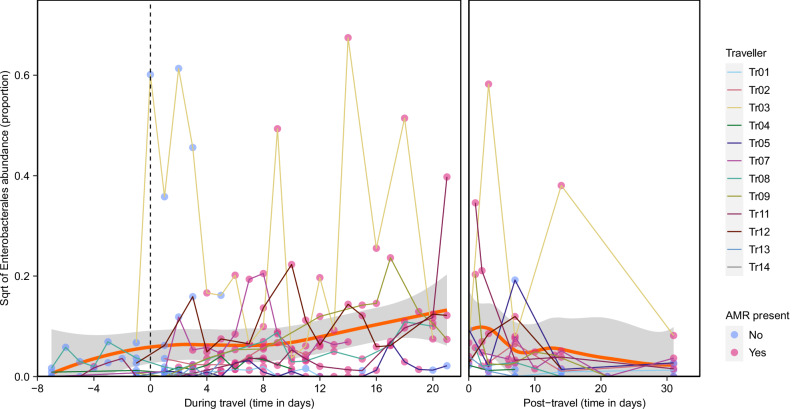
Temporal dynamics of Enterobacterales abundance in travellers. Square-root-transformed (sqrt) relative abundance is shown over time (pre-, during-, and post-travel) for individual participants. The orange line depicts a fitted Locally Estimated Scatterplot Smoothing (LOESS) curve. Dots connecting the lines represent individual samples and are coloured pink when at least one ARG was detected and blue when the sample was negative for all ARGs. The standard error (SE) confidence band is displayed in grey.

Finally, we evaluated the shifts in the abundance of dominant microbial taxa in the travellers’ gut microbiota, focusing on pre-travel and during travel samples. Overall, we observed a statistically significant increase of Enterobacterales during travel (LinDA; adjusted *P* < 0.001; Fig. 5), which coincided with the acquisition of one or more ARGs (*bla*_CTX-M_, *qnrS* or *qnrB*). Group level analysis of AMR genes across acquisition phases revealed a significant increase in Enterobacterales abundance following acquisition of *qnrS* compared with samples that remained *qnrS* negative (paired Wilcoxon rank sum test, adjusted *P* = 0.04; Supplementary Fig. 20). A similar trend was observed following acquisition of *bla*_CTX-M_ and *qnrB* (adjusted *P* = 0.08 for both), indicating a consistent association between acquisition of these resistance genes and expansion of Enterobacterales within the gut microbiota. Following travel, Enterobacterales abundance showed a decreasing trend towards prior to travel levels (LinDA, *P* = 0.026, adjusted *P* = 0.502; Fig. 5).

In addition, we observed statistically significant decreases in the abundances of *Oscillospirales*, Clostridia vadinBB60 group, *Lachnospirales* and *Bifidobacteriales* during travel (LinDA; all *P* < 0.05, adjusted *P* < 0.2; Supplementary Fig. 21). Notably, these decreases occur in parallel with the acquisition of ARGs as well as with a decrease in microbial diversity during the initial 5 days of travel (Figs. 1A and 4).

## Discussion

Here, we present the first study employing daily sampling to elucidate the temporal dynamics of gut microbiota composition and AMR gene acquisition during intercontinental travel and showed that compositional shifts and acquisition of AMR genes can occur within days of arrival at the travel destination. While species diversity remained generally stable across study periods, the results showed an initial decline within the first 5 days of travel, which coincided with the earliest detection of AMR genes, as well as overall decreases in several bacterial taxa. This early period appeared to be critical for microbial shifts, suggesting a narrow window during which travellers are most susceptible to acquiring resistant organisms.

Previous research has established that travel can influence gut microbiota and/or facilitate the acquisition of AMR genes^13–15,20–23^. However, these studies often relied on pre- and post-travel sampling, potentially overlooking rapid and transient microbial changes observed from our results. By implementing a daily sampling regimen, our study provides a granular view of the gut microbiota’s responsiveness to external stimuli during travel. This approach allows for the detection of short-lived microbial fluctuations that might be missed with less frequent sampling intervals.

A previous study by Kantele et al.^24^ investigated the acquisition dynamics of ESBL-producing Gram-negative bacteria in 20 European travellers visiting Laos and highlighted the transient nature of colonisation and the rapid acquisition of multiple resistant strains during travel. This aligns with our findings of rapid AMR gene acquisition, increased abundance of Enterobacterales during travel, and the transient detection of AMR genes over time. Importantly, whereas Kantele et al. relied on culture-based isolate analyses, our study used targeted qPCR to monitor ARG dynamics, enabling sensitive detection of resistance determinants independent of bacterial isolation. The intermittent detection patterns observed for *qnrS*, *qnrB* and *bla*_CTX-M_ likely reflect dynamic fluctuations in both relative abundance within the microbial community and overall bacterial load, rather than true acquisition or complete loss events. Together, these findings support the notion that travel-associated resistome dynamics are highly transient and complex, and that studies limited to pre- and post-travel sampling may underestimate the temporal variability of AMR gene carriage. In addition, the initial decline of commensal bacteria combined with the presence of AMR genes during the early period of travel suggests that an altered colonisation resistance due to microbiota perturbations caused by travel is potentially involved in AMR acquisition risk. Future research involving this critical early window is necessary to uncover the role of microbial perturbations in resistome dynamics.

Interestingly, in every participant included in this study, at least one of the targeted ARG was detected, indicating that ARG acquisition during travel to regions in Asia prevalent with resistant bacteria is common and even short visits to high-risk environments pose a substantial risk for AMR gene uptake. While precise prevalence rates cannot be inferred due to the limited cohort size, these findings are consistent with earlier studies^23,25,26^. The *qnrS* gene has been reported at high frequencies in bacterial isolates and fecal metagenomes from several Asian countries, reinforcing the notion that regional AMR burden directly influences ARG acquisition in travellers^24,25^.

Moreover, genes from the *bla*_CTX-M_ family were detected in travellers to India, South Korea, and Malaysia. These genes are known to be widespread in Asia, and previous studies have shown that a significant proportion of travellers returning from South and Southeast Asia carry multidrug-resistant Enterobacterales, often harbouring *bla*_CTX-M_ genes^25,26^. Notably, we detected *bla*_CTX-M_ in hand skin swabs of one of the travellers to India, while the fecal sample of the same individual remained negative. This suggests that although colonisation of the gut microbiota may not always occur or remain undetected, there can still be significant exposure to ARGs through environmental contact. This emphasises the importance of hand hygiene as a preventive measure.

Our cohort of only 11 participants may be considered a limitation due to not being able to fully capture the variability of gut microbiota across diverse populations. In addition, some practical challenges, such as variations in individual bowel habits or low biological material during fecal swabbing, may have led to missing samples on certain days as well as occasional failures in AMR gene detection due to low DNA yield, respectively. The latter could explain the presence of intermittent negative samples interspersed among ARG-positive samples. This potentially leads to an underestimation of how quickly and frequently ARGs are acquired and highlights the need for careful and consistent sample collection methods in future studies. In addition, longer and more standardised follow-up periods will be necessary to determine whether travel-acquired ARGs become durably established or are largely transient, and to assess the longer-term trajectory of microbiome recovery. Lastly, this study is observational in nature and therefore focuses on identifying associations rather than causal relationships between travel, microbiome shifts, and ARG acquisition. While key potential confounders, including detailed dietary intake, environmental exposures, and pathogen presence, were not systematically assessed, these factors represent important opportunities for future studies to further refine and extend the mechanistic understanding of the observed patterns.

In conclusion, the daily sampling methodology employed in this study offers a novel and detailed perspective on the gut microbiota’s dynamics during international travel. The identification of the initial five days post-arrival as a critical period for microbial shifts and AMR gene acquisition highlights the need for timely preventive measures. Future research should build upon this approach to further explore the factors influencing early microbiota changes and AMR acquisition during travel and to develop targeted interventions aimed at reducing the risk of AMR dissemination associated with global travel.

## Methods

### Study population and sample collection

In this prospective cohort study, a total of 11 healthy Dutch volunteers who were planning to travel outside of Europe and had not used antibiotics in the previous 3 months were recruited at the Maastricht University Medical Centre. The participants were asked to collect fecal swabs before, during and after an international trip to Asia. Fecal swabs were collected multiple times during the week prior to travel, on each day that defecation occurred during travel, and again at 1 and 3 days, as well as at 1, 2 and 4 weeks after returning from travel. For some travellers, additional samples were collected at 3 and/or 6 months post-travel.

To explore whether carriage of AMR bacteria on the skin preceded intestinal colonisation, we additionally collected swabs from the palm and fingers of the dominant hand at any given point during the day, preferably consecutively during the first 3–7 days of travel in a subgroup of six participants. All swabs were immediately stored in DNA/RNA Shield (Zymo Research, Irvine, USA) to ensure the stability of the metagenome during unrefrigerated transport.

All participants also provided information on travel dates and destinations, gastrointestinal infections as well as antibiotic use during the study period.

### Ethics declaration

All participants provided written informed consent. The study was in accordance with the Helsinki Declaration and was assessed by the Maastricht University Medical Centre medical ethics committee. The medical ethics committee has declared that this study does not fall under the Medical Research Involving Human Subjects Act (METC 2018-0701).

### Sample processing and DNA extraction

The fecal and hand swabs (DNA/RNA Shield) were stored at ambient temperature during the participants’ travels. DNA/RNA Shield stabilises DNA and RNA at ambient temperature, inactivates pathogens and is validated for diverse sample types such as blood, feces and swabs. They were subsequently stored at −20 °C in the lab until DNA extraction. Identical sampling methods were employed for all subjects. The extraction of DNA was performed as described previously^27^. Briefly, 200 µl of DNA/RNA Shield, in which the swabs were suspended, was added to a 2-mL vial containing 0.5 g of 0.1 mm zirconia/silica beads (BioSpec, Bartlesville, OK, USA), four glass beads (3.0–3.5 mm)(BioSpec) and 1.2 ml of lysis buffer from the PSP Spin Stool Kit (Stratec Molecular, Berlin, Germany). Samples were disrupted in a MagNA Lyser device (Roche, Basel, Switzerland) in three cycles of 1 min at a speed of 5500 rpm. Subsequently, DNA was isolated from the samples using the PSP Spin Stool Kit, according to the manufacturer’s instructions. In addition to the samples, negative sampling and four DNA isolation controls, as well as two ZymoBIOMICS® Microbial Community Standard (D6300; Zymo Research, Irvine, CA, USA) mocks as positive control were included during DNA isolation and downstream sample handling.

### 16S rRNA gene sequencing and pre-processing

Amplification of hypervariable V4 region from the 16S rRNA gene was performed using primer pairs 515 F (forward: 5’-GTGCCAGCMGCCGCGGTAA-3’) and 806 R (reverse: 5’-(GGACTACHVGGGTWTCTAAT-3’)^27,28^. Following amplification, PCR products from triplicate reactions were combined to reduce variability. Subsequently, purification was performed using the AMPure XP magnetic bead system (Beckman Coulter, Massachusetts, USA) and eluted in 25 µl of 1× low TE buffer (10 mM Tris-HCl, 0.1 mM EDTA, pH 8.0). Quantification of amplicons was determined using the Quant-iT™ PicoGreen® dsDNA Assay Kit (Invitrogen, New York, USA) with a Victor3 Multilabel Counter (Perkin Elmer, Waltham, USA). Equal amounts of amplicons from each sample were pooled to ensure uniform representation in the sequencing library. Paired-end sequencing was performed on an Illumina MiSeq platform (Illumina, San Diego, California, USA), utilising the MiSeq Reagent Kit v3 (2 × 250 cycles) and incorporating 10% PhiX.

The sequencing data was pre-processed, using an in-house pipeline based upon DADA2, consisting of reads filtering, identification of sequencing errors, dereplication, inference and removal of chimeric sequences^29^. The final length of the forward and reverse reads was 240 and 160 nucleotides, respectively. The standard filtering setting was used with the enforcement of a maximum of 2 expected errors per-read. Subsequently, the reads were dereplicated to remove identical sequences and inferred and merged to obtain the full denoised sequences. Sequences with forward and reverse reads overlapping by at least 12 bases, and those that were identical to each other in the overlap regions were aligned together. To assign taxonomy, DADA2 was used to annotate up to the genus level using the database SILVA 138 version 2^30^. Decontam was applied for the identification and removal of contaminant Amplicon Sequence Variants (ASVs) using the “either” option, which integrates both the prevalence and frequency statistical approaches to detect contamination in marker-gene and 16S rRNA gene sequencing datasets^31^. Data were expressed as ASV. Samples with a total read count of <10,000 were excluded from further analysis.

### Real-time PCR assays

Real-time PCR was performed to detect the following ARGs: *qnrB, qnrS* and *bla*_CTX-M,_ as described previously^32^. These targets were selected a priori based on their clinical relevance, documented global dissemination, and previously demonstrated increased abundance and acquisition frequency after international travel in several Dutch travel cohorts^32,33^. Details on primers and probes are available in the Appendix (Supplementary Table 1).

The *qnrB, qnrS* and *bla*_CTX-M_ were amplified on an Applied Biosystems QuantStudio 5 Real-Time PCR System using TaqPath™ qPCR Master Mix (Applied Biosystems) with cycling conditions of 15’ at 95 °C, followed by 45 cycles of 15” at 95 °C and 60” at 60 °C. The probes to detect *bla*_*CTX-M*_ groups 1 and 2 were combined in a single multiplex reaction, while a separate qPCR reaction was used to detect *bla*_*CTX-M*_ group 9. Nuclease-free water was used as negative control while positive controls included pGEM-T Easy (Promega Corporation) plasmids containing the ARGs *qnrS*, *qnrB* and *bla*_*CTX-M*_ groups 1, 2 and 9.

To normalise for the different amounts of bacterial DNA in the samples, a 500 bp fragment 16S rDNA amplification was used. The 16S rDNA was amplified on a MyiQ™ Single-Colour Real-Time PCR Detection System (BioRad, Hercules, CA, USA) in 25 μl volume containing 12.5 μl iQ™ SYBR® Green Supermix (BioRad), 5 μl template DNA and 300 nM of both forward (5’-CCTACGGGNGGCWGCAG-3’) and reverse (5’-GACTACHVGGGTATCTAATCC-3’) primers^28^. The cycling conditions were as follows: 3’ at 95 °C, followed by 35 cycles of 15” at 95 °C, 20” at 55 °C and 30” at 72 °C, followed by a melting curve analysis. The amplification efficiency was determined to be 103% by performing triplicate measurements of standard curves of a pGEM-T easy plasmid construct (Promega Corporation) harbouring the targeted 16S rDNA sequence.

### Statistical analysis

The study utilised a convenience sample; therefore, power calculations were not performed. All downstream analysis was performed in R (v.4.1.3). Alpha and beta diversity were calculated using R packages ‘phyloseq (v1.38)’ and ‘microviz (v0.10.1)’ while plots were constructed in ‘microviz’ and ‘ggplot2 (v3.4.3)’. To minimise the impact of spurious low-abundance features, ASVs representing <0.01% of the total reads across all samples were excluded from downstream analyses.

To account for scale invariance and the compositional characteristics of sequencing-derived count data, we estimated the relative abundance profiles by modelling the data using a Dirichlet distribution, implemented with 128 Monte Carlo simulations via the ALDEx2 package (v1.24.0). The resulting values were subjected to a centred log-ratio (clr) transformation to mitigate the constraints inherent in compositional microbiome data^34^.

Pairwise dissimilarity between samples was quantified using the Aitchison distance, which represents the Euclidean distance between clr-transformed vectors, and was computed using the vegan package (v2.6-4). This distance metric served as the basis for evaluating beta diversity. Sample ordination was performed through PCA on the Aitchison distance matrix. The ordination plots were further integrated with metadata using the microViz package, and final visualisations were produced with ggplot2.

To model the effect of travel on gut microbial diversity, generalised linear mixed-effects models (GLMMs) with a gamma distribution and log link were fitted by maximum likelihood using the glmmTMB package (v1.1.7). To assess the effect of travel on gut compositional dissimilarity, linear mixed-effects models (LMMs) were fitted by maximum likelihood using the lmer function in R. Models were specified to evaluate changes in α-diversity and β-diversity, with time period (pre-travel, during travel, post-travel) included as a fixed effect, and traveller included as a random effect. In addition, LMMs were used to assess the association between *bla*_CTX-M_ acquisition and Shannon diversity, and between the presence of *qnrB, qnrS* or *bla*_CTX-M_ and Shannon diversity. Post hoc pairwise comparison between different time periods (e.g., Pre-travel vs during travel) was performed on fitted models (for α-diversity and β-diversity) using the emmeans package (v1.10.5), with Tukey’s Honest Significant Difference (HSD) adjustment for multiple testing. Significance was set at *P* < 0.05 (Tukey-adjusted). Differences in AMR gene positivity across travel periods were assessed using Friedman tests on traveller-level percentage positivity (defined as the proportion of positive samples per traveller and period), including only travellers with complete observations across all periods. Post hoc pairwise comparisons were performed using paired Wilcoxon signed-rank tests restricted to complete pairs. Shannon diversity was analysed relative to the AMR gene acquisition phase (pre-acquisition, post-acquisition positive and post-acquisition negative), with traveller-level mean Shannon diversity calculated by averaging samples within each phase. Differences between phases were assessed using paired Wilcoxon signed-rank tests, including only complete pairs. Enterobacterales abundance was analysed relative to AMR gene acquisition phase, with traveller-level mean abundances calculated per phase and square root transformed prior to analysis; phase-wise differences were evaluated using paired Wilcoxon signed-rank tests restricted to complete pairs. For all pairwise comparisons described above, *p* values were adjusted using the Benjamini–Hochberg false discovery rate (FDR) correction, and adjusted *P* values < 0.05 were considered statistically significant.

Differential abundance analysis was performed using Linear Models for Differential Abundance (LinDA, R package v0.1.0)^35^, which allows modelling of longitudinal data by accounting for repeated measurements when identifying microbial taxa associated with travel and post-travel. The taxonomic count table at the Order level was used as the response matrix. Orders present in less than 60% of the samples were excluded to reduce the influence of sparsely represented taxa and enhance the robustness of the analysis (prev.cut = 0.6). In the model formula, time was included as a continuous variable while also adjusting for country travelled, age, sex, as well as inter-individual variability by specifying subjects as the random effects. *P* values were corrected for multiple hypotheses using the Benjamini–Hochberg method, and significance was set at FDR < 0.20.

## Supplementary information

Supplementary tables and figures

Supplementary files

## Data Availability

16s rRNA gene sequence data will be made available publicly with publication at the European Nucleotide Archive under Bioproject PRJEB96287. Associated metadata are not publicly available due to privacy considerations, but can be obtained from the authors upon reasonable request.
