# Supplementary material for: Daily sampling reveals rapid microbiota alterations and antimicrobial resistance gene acquisition during intercontinental travel
Source: NPJ Biofilms Microbiomes. 2026 Apr 7;12:108. doi: 10.1038/s41522-026-00977-x (PMC13234331; doi:10.1038/s41522-026-00977-x)
Supplement: Supplementary file 1 — Supplementary tables and figures [file 41522_2026_977_MOESM1_ESM.docx]

**Appendix 1**

**Daily sampling reveals rapid microbiota alterations and antimicrobial resistance gene acquisition during intercontinental travel**

**Supplementary Table 1.** PCR primer/ probe sequences for AMR genes identification.

| **Primer/ probe** | **Sequence* 5’ -3’** | **Target** | **Reference** |
| --- | --- | --- | --- |
| 16S_341_F | CCTACGGGNGGCWGCAG | 16S rDNA | 1, 2 |
| 16S-805_R | GACTACHVGGGTATCTAATCC |  |  |
| *CTX-M*-F | ATGTGCAGYACCAGTAARGTKATGGC |  | 2, 3 |
| *CTX-M*-R | ATCACKCGGRTCGCCNGGRAT |  |  |
| *CTX-M*-Probe-1 | JOE-CCCGACAGCTGGGAGACGAAACGT-BHQ-1 | CTX-M-1 |  |
| *CTX-M*-Probe-2 | 6-FAM-CAGGTGCTTATCGCTCTCGCTCT GTT-BHQ-1 | CTX-M-2 |  |
| *CTX-M*-Probe-9 | JOE-CTGGATGCACTGAACCTACGCTGA-BHQ-1 | CTX-M-9 |  |
| qnrB_F | CAGATTTYCGCGGCGCAAG | qnrB | 2, 4 |
| qnrB_R | TTCCCACAGCTCRCAYTTTTC |  |  |
| qnrB_probe | 6FAM-CGCACCTGGTTTTGYAGYGCMTATATCAC-BHQ1 |  |  |
| qnrS_F | TCAAGTGAGTAATCGTATGTA | qnrS | 2, 4 |
| qnrS_R | GTCTGACTCTTTCAGTGAT |  |  |
| qnrS_probe | 6FAM-CCAGCGATTTTCAAACAACTCAC-BHQ1 |  |  |

1. Klindworth A, Pruesse E, Schweer T, Peplies J, Quast C, Horn M, Glöckner FO. Evaluation of general 16S ribosomal RNA gene PCR primers for classical and next-generation sequencing-based diversity studies. Nucleic acids research. 2013 Jan 1;41(1):e1-.

2. von Wintersdorff CJ, Penders J, Stobberingh EE, et al. High rates of antimicrobial drug resistance gene acquisition after international travel, The Netherlands. Emerging infectious diseases 2014; 20(4): 649.

3. Kim SM, Kim HC, Lee SW. Characterization of antibiotic resistance determinants in oral biofilms. The Journal of Microbiology. 2011 Aug;49(4):595-602.

4. Vien LT, Minh NN, Thuong TC, Khuong HD, Nga TV, Thompson C, Campbell JI, de Jong M, Farrar JJ, Schultsz C, van Doorn HR. The co-selection of fluoroquinolone resistance genes in the gut flora of Vietnamese children.

**Supplementary Figs. 1-10. Microbial composition and diversity profiles of traveller subjects over time**. Microbial composition and diversity in traveller subjects over time. Principal component analysis (PCA) of the gut microbiota coloured by time period (prior to travel, during travel and post-travel). Shannon diversity is plotted over time, with collection points coloured by the presence of antimicrobial resistance genes (ARGs) detected at each time point. Bar plots show the relative abundance of the top 10 bacterial genera per time period with bars positioned chronologically by sampling time points within each period.

**
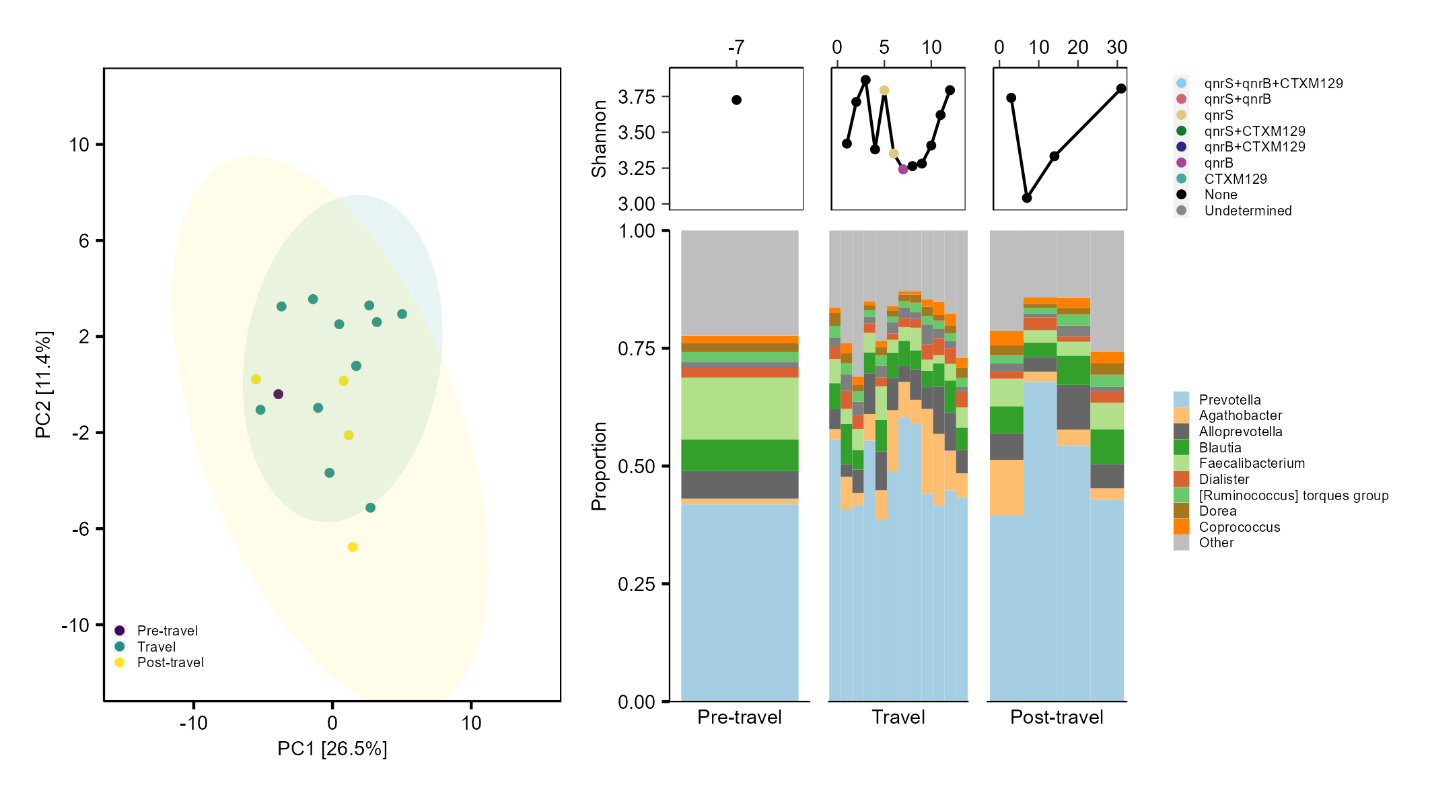
Supplementary Fig. 1 Traveller Tr01**

**
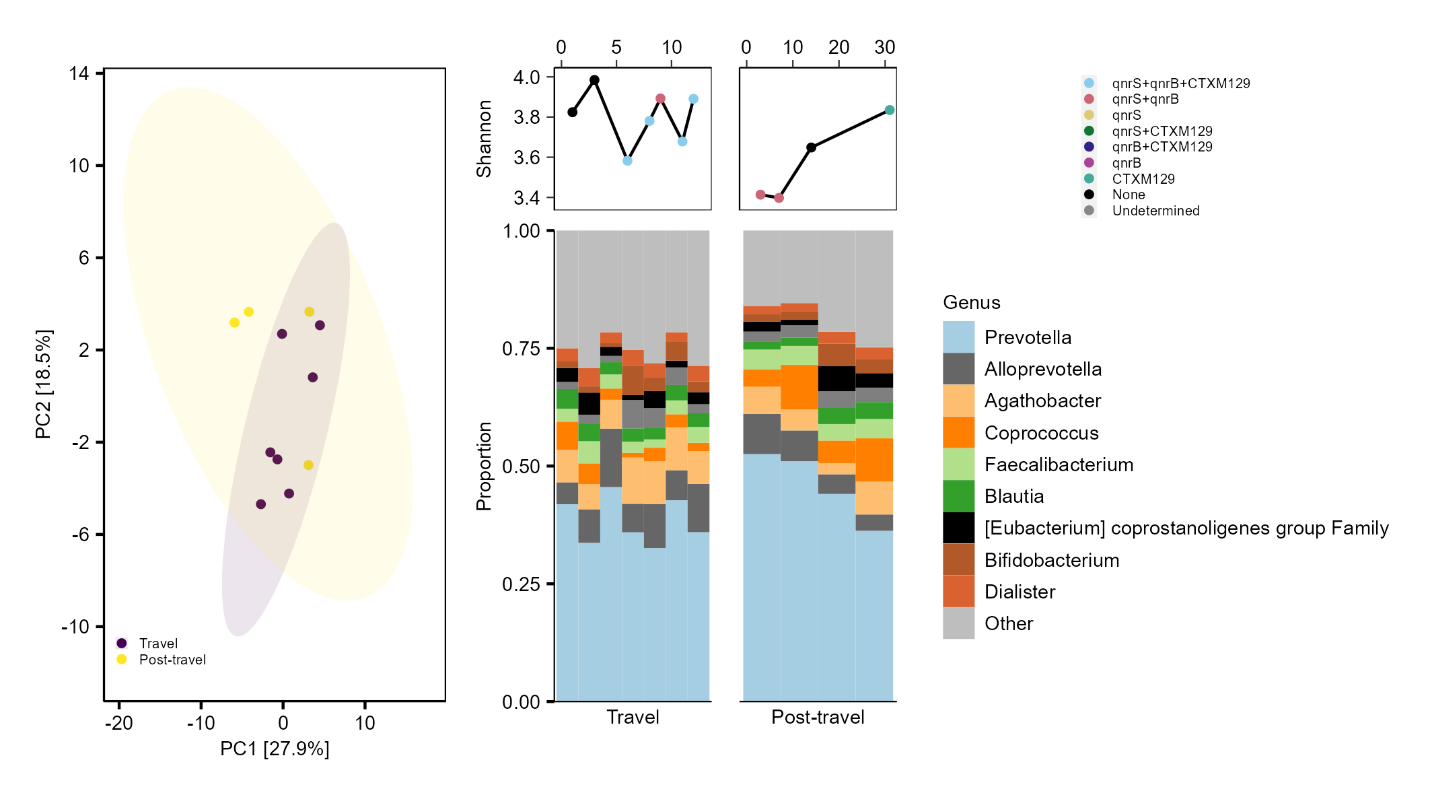
**

**Supplementary Fig. 2 Traveller Tr02**

**
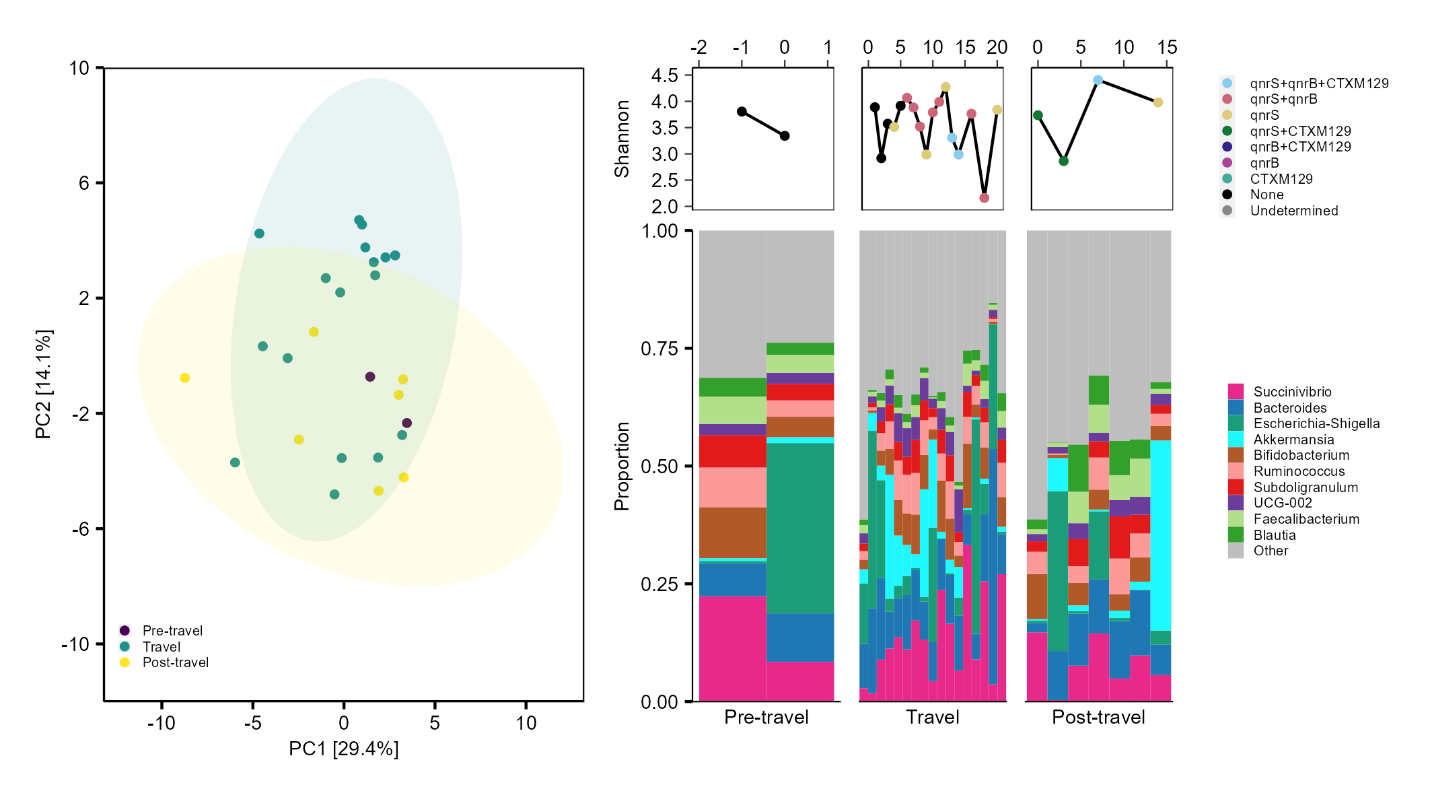
Supplementary Fig. 3 Traveller Tr03**

**
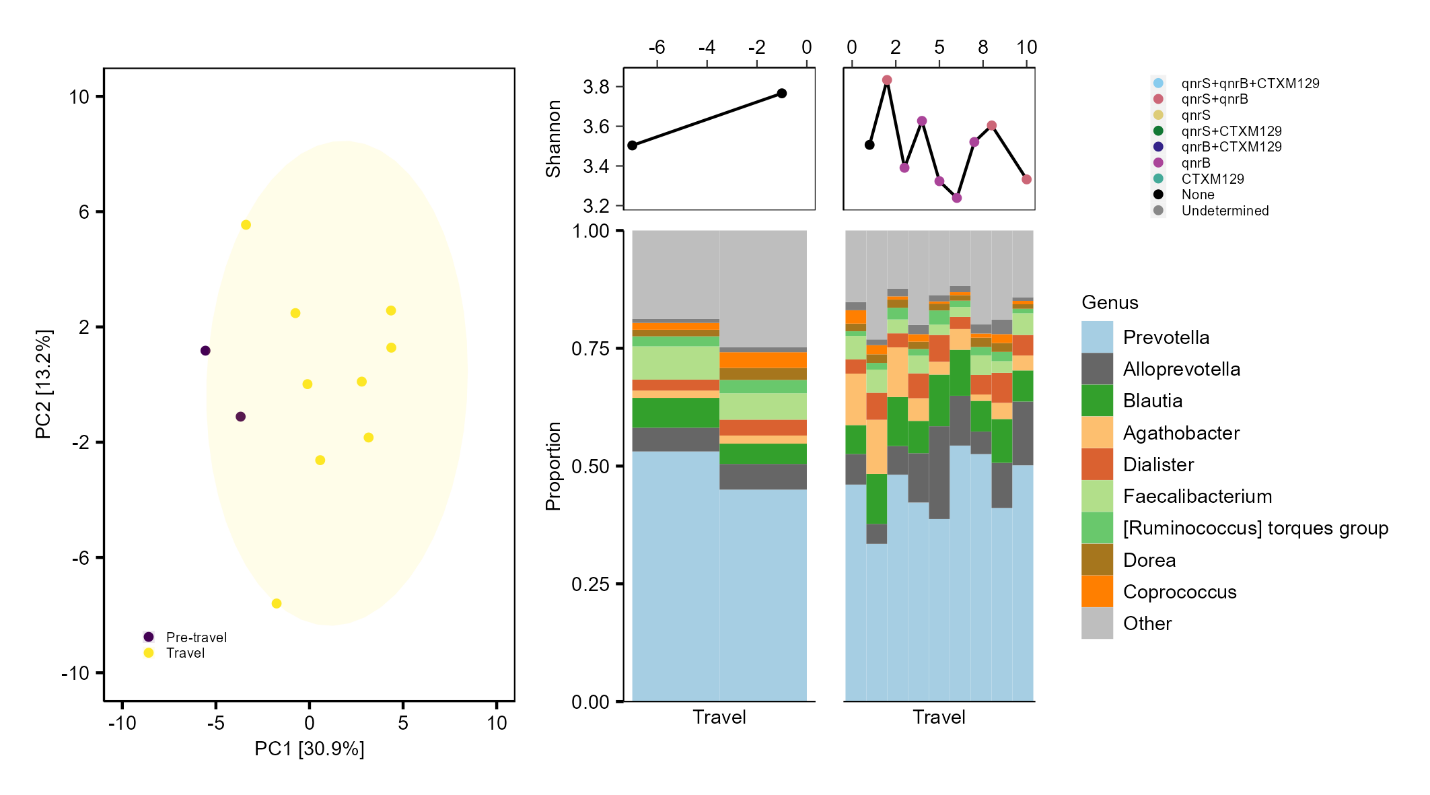
 Supplementary Fig. 4 Traveller Tr04**

**
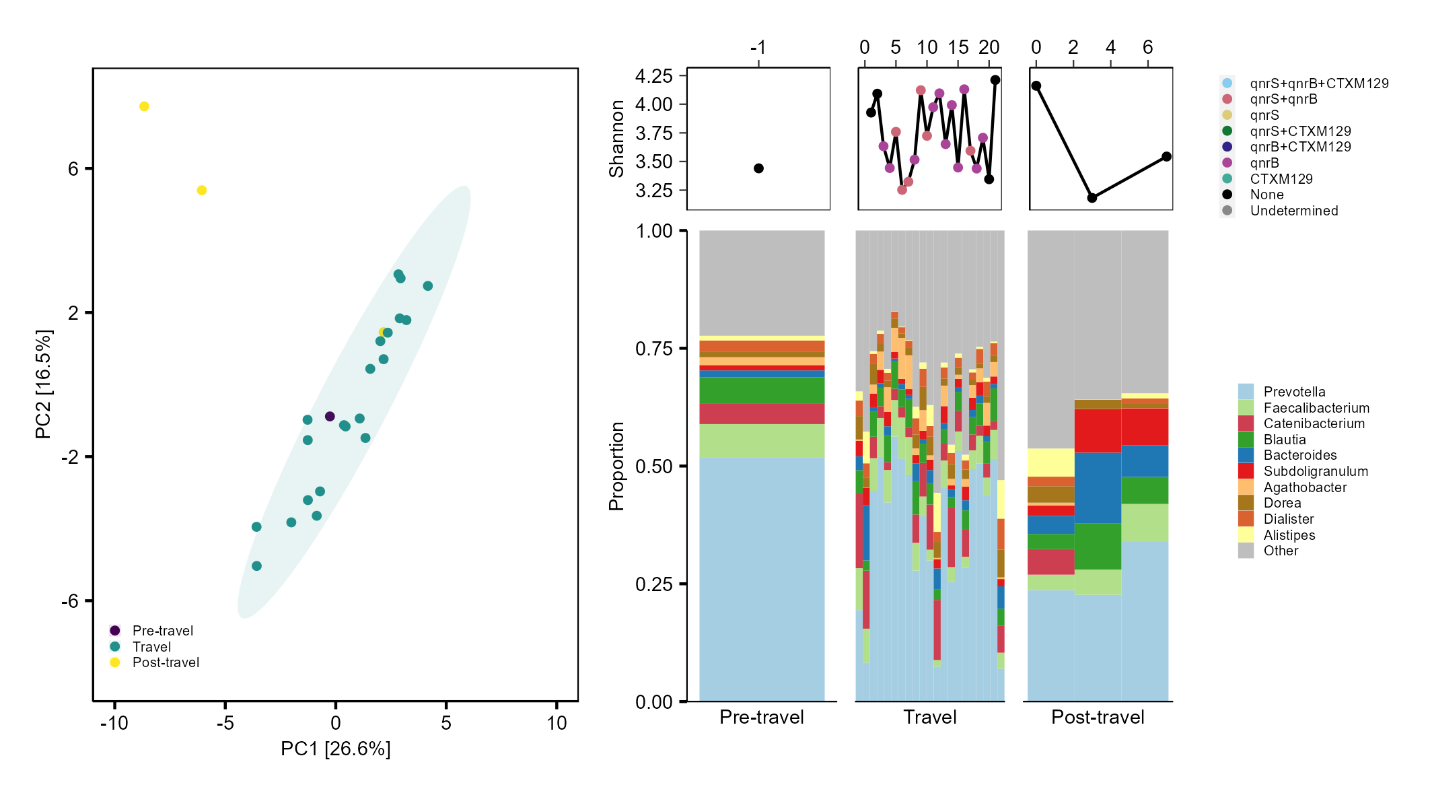
Supplementary Fig. 5 Traveller Tr05**

**
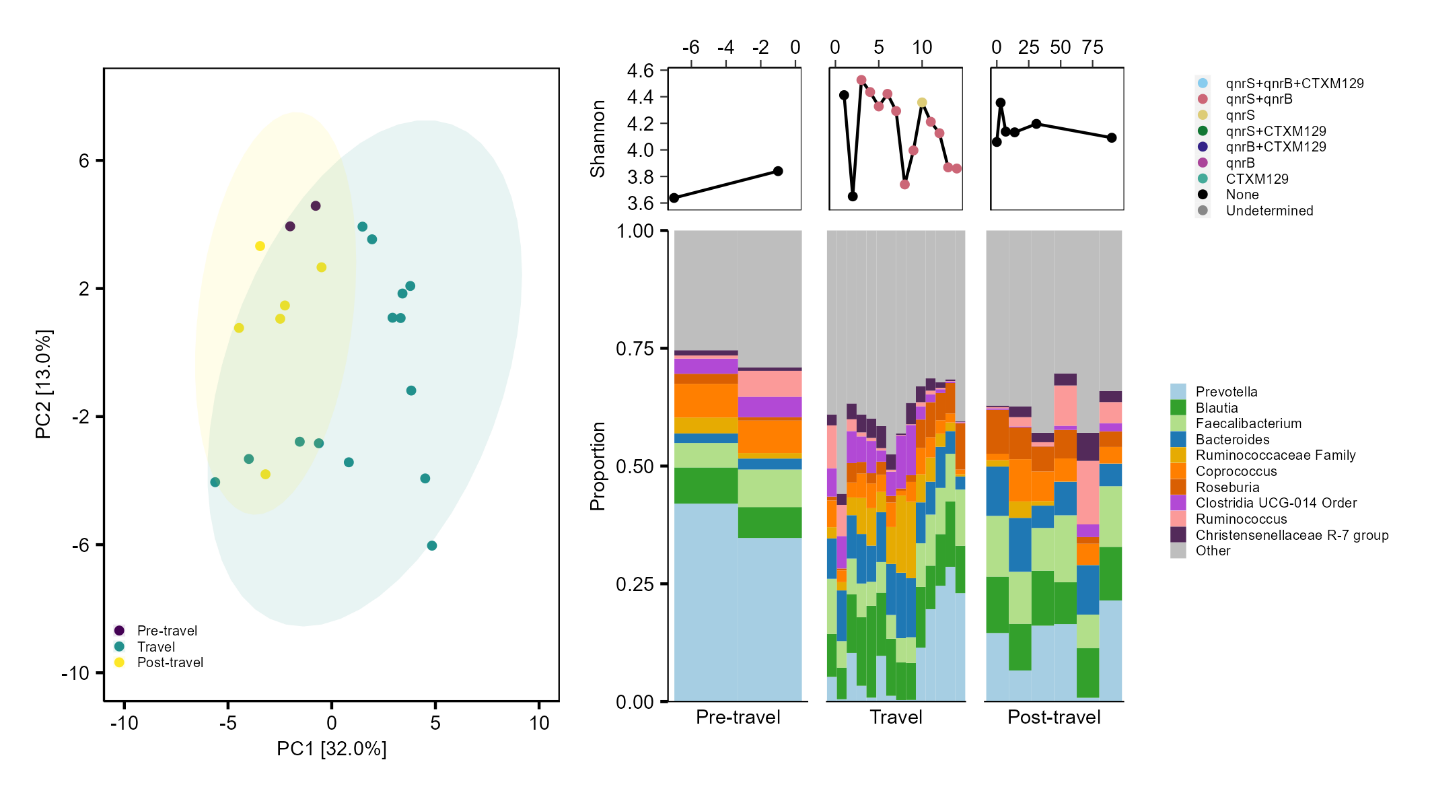
Supplementary Fig. 6 Traveller Tr07**

**
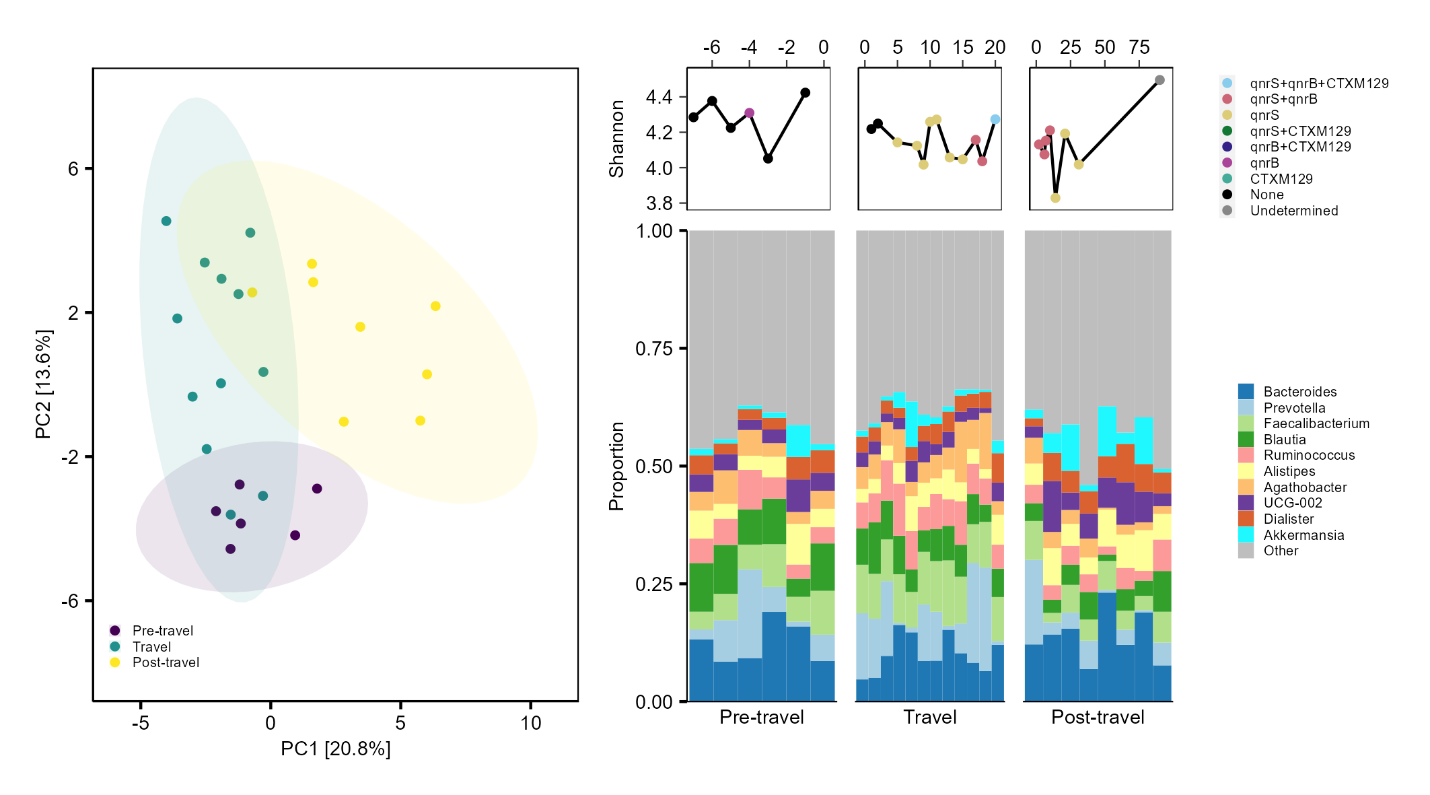
Supplementary Fig. 7 Traveller Tr08**

**
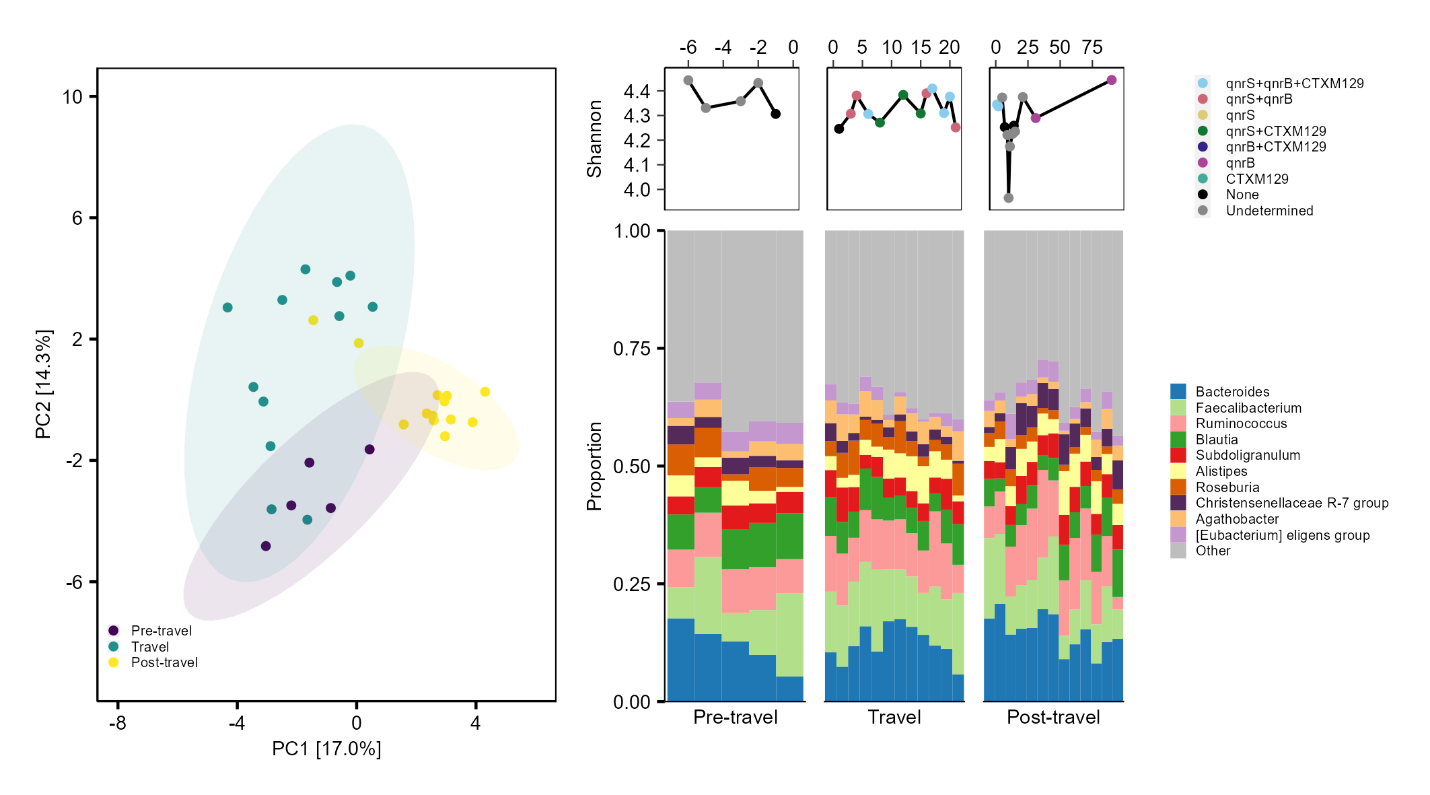
Supplementary Fig. 8 Traveller Tr09**

**
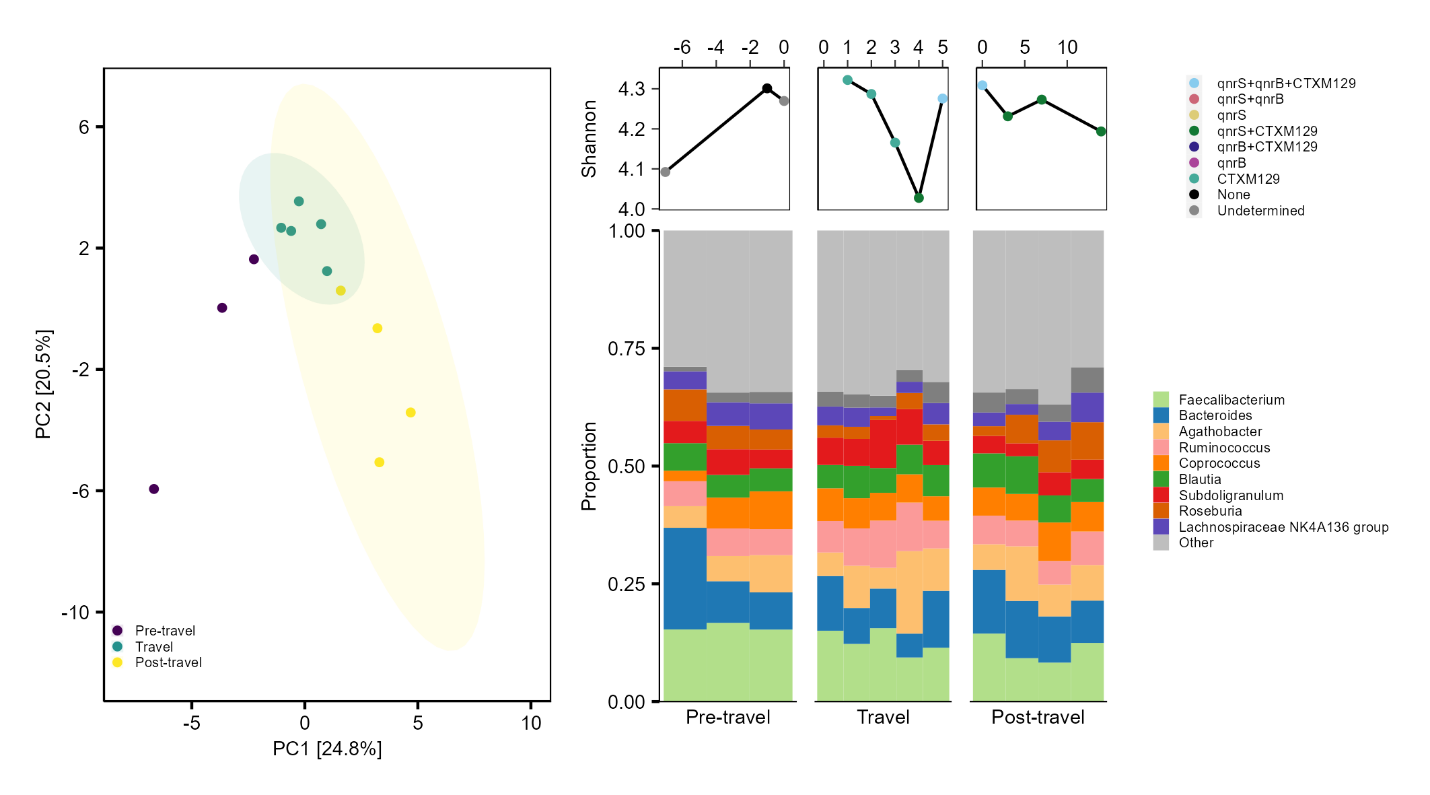
Supplementary Fig. 9 Traveller Tr13**


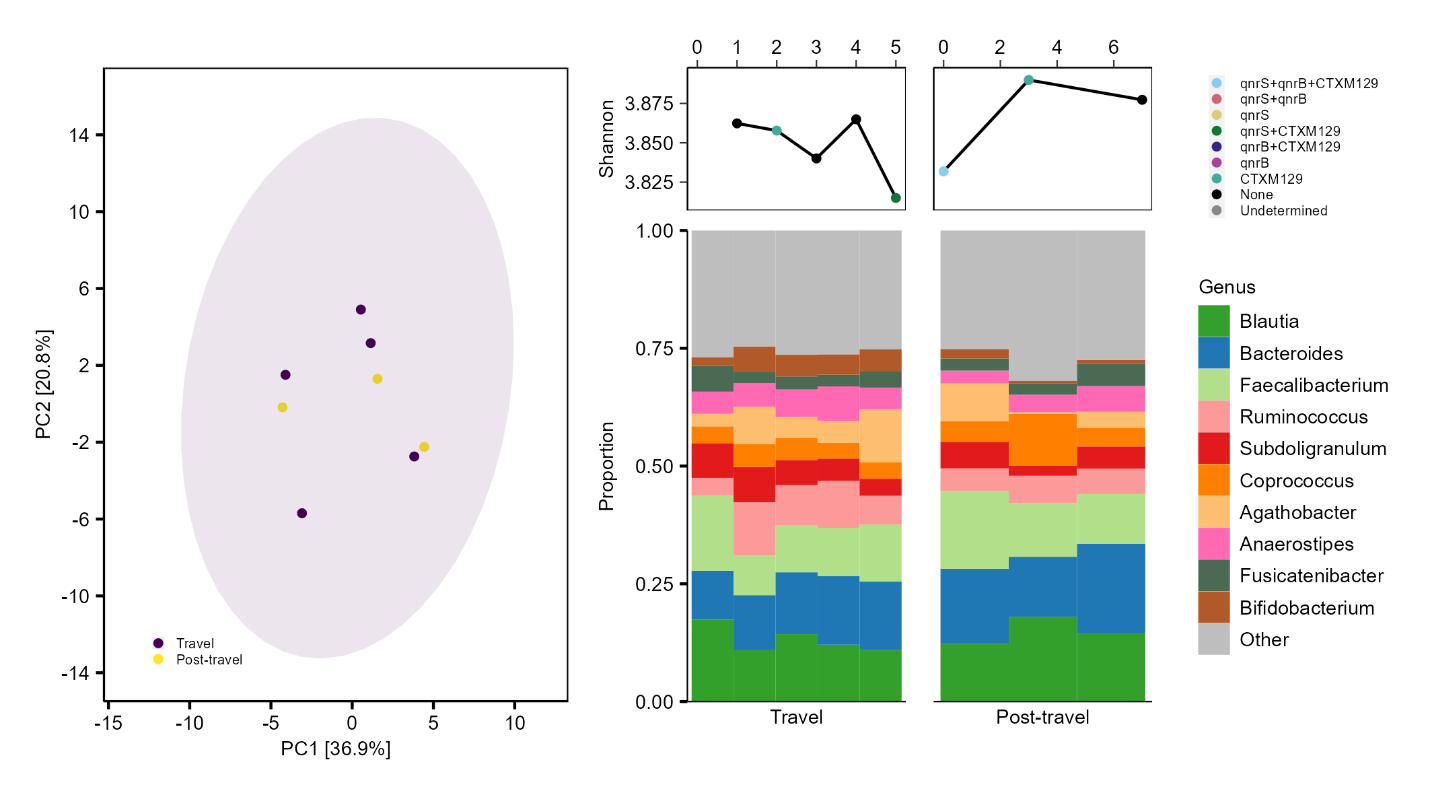
**Supplementary Fig. 10 Traveller Tr14**


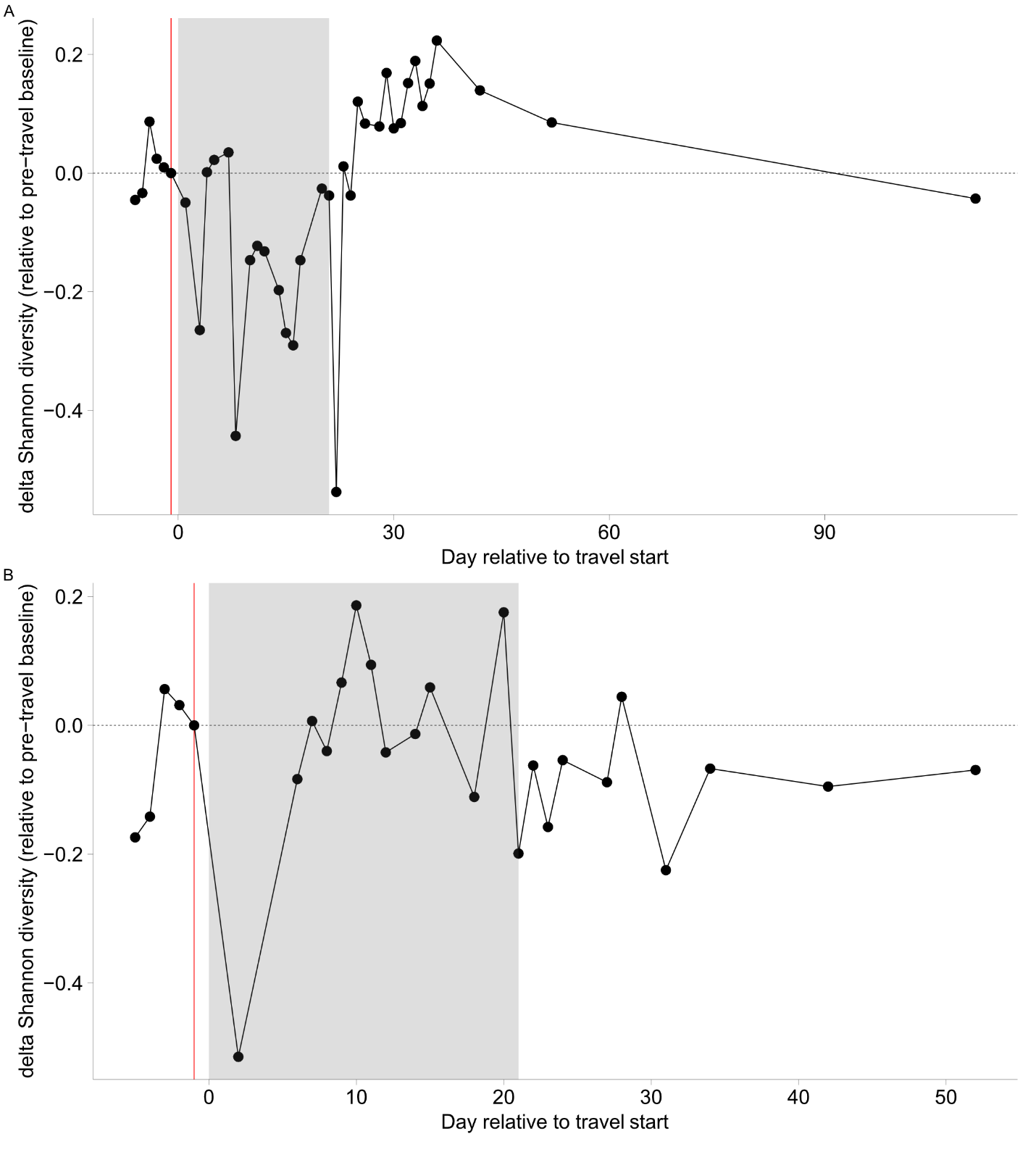
**Supplementary Fig.11 Longitudinal changes in Shannon diversity relative to travel start.** (A) Shannon diversity index for traveller Tr12 across pre-travel, travel, and post-travel periods. (B) Shannon diversity index for traveller Tr11 across the same periods. Values are plotted relative to day of sampling, with the individual pre-travel baseline defined as the final pre-travel time point (vertical red line, day −1). The shaded region indicates the travel period. Lines connect consecutive sampling time points and points represent individual faecal swab samples. Horizontal dashed line denotes zero change from baseline.


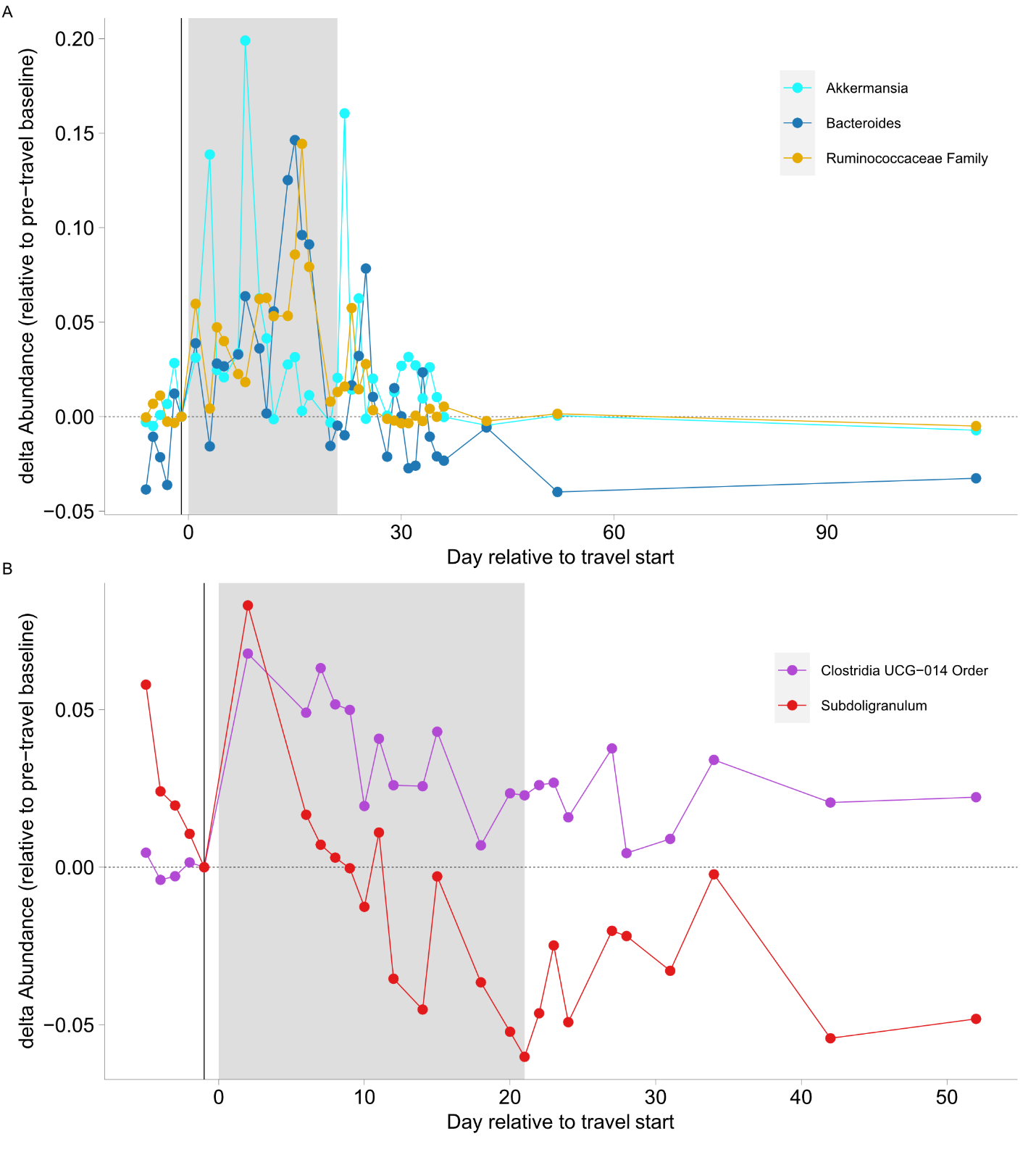
**Supplementary Fig. 12 Longitudinal dynamics of gut bacteria relative to travel start**. (A) Relative abundance trajectories of Bacteroides, Akkermansia, and members of the Ruminococcaceae family for traveller Tr12 across pre-travel, travel, and post-travel periods. (B) Relative abundance trajectories of Clostridia UCG-014 Order and Subdoligranulum for traveller Tr11. Delta abundance represents the change relative to the individual pre-travel baseline, defined as the final pre-travel time point (vertical black line, day −1). The shaded region indicates the travel period. Lines connect consecutive sampling time points and points represent individual samples. Horizontal dashed line denotes zero change from baseline.

**
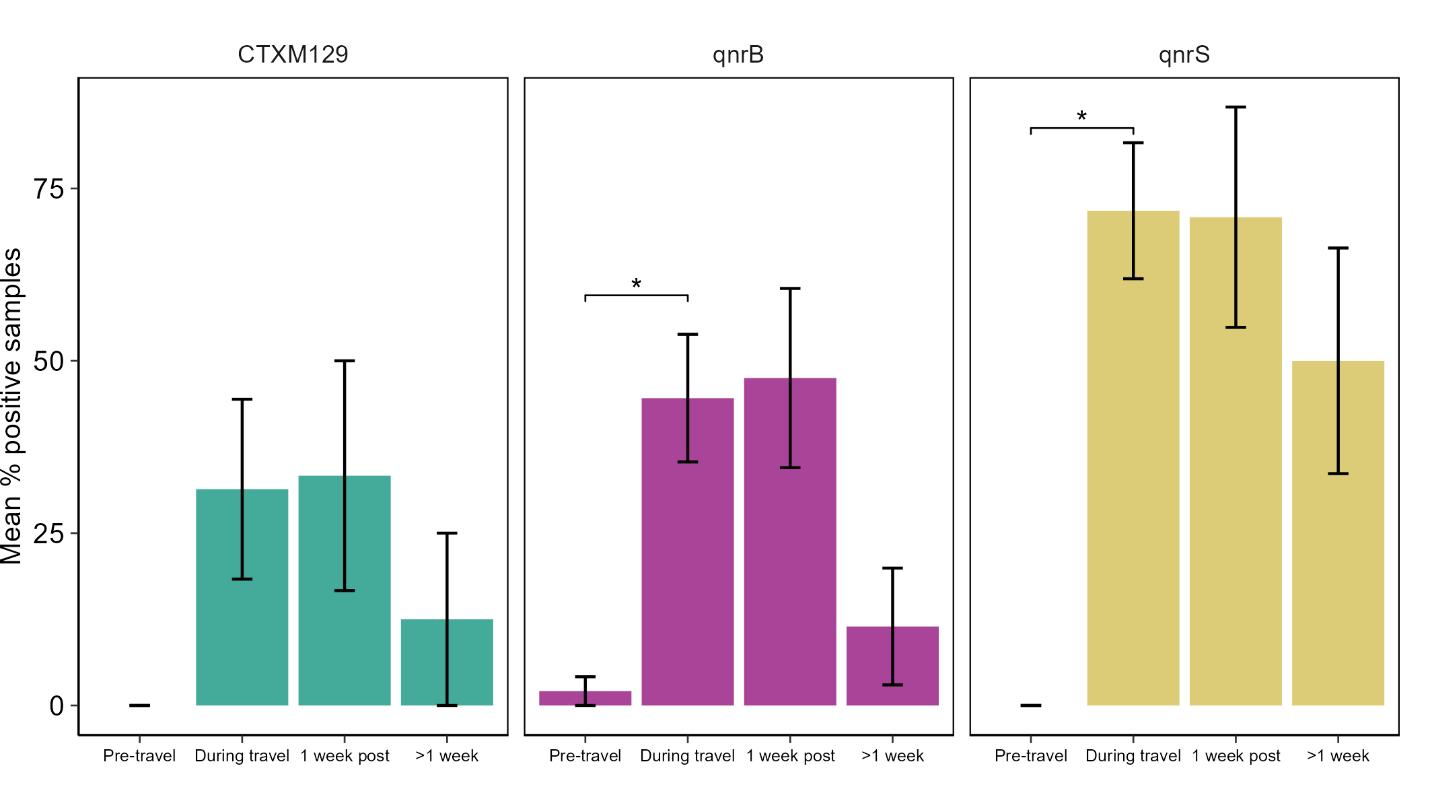
**

**Supplementary Fig. 13 Detection dynamics of antimicrobial resistance genes in faecal swabs across travel-associated time periods.** The bar plot shows the mean percentage of positive swabs for three antimicrobial resistance genes (qnrS = *qnrS*, qnrB = *qnrB*, and CTXM129 = *bla*_CTX-M_ group 1, 2 and 9) across four time periods: pre-travel, during travel, 1 week post-travel, and >1 week post-travel. For each traveller, gene-specific positivity rates were calculated per time period and averaged across travellers to obtain group-level means. Bars show mean percentages of positive samples per gene and period, with error bars indicating the standard error. Asterisks denote statistically significant pairwise comparisons following Friedman test with post hoc paired Wilcoxon tests and Benjamini–Hochberg correction (* adjusted p < 0.05).

**
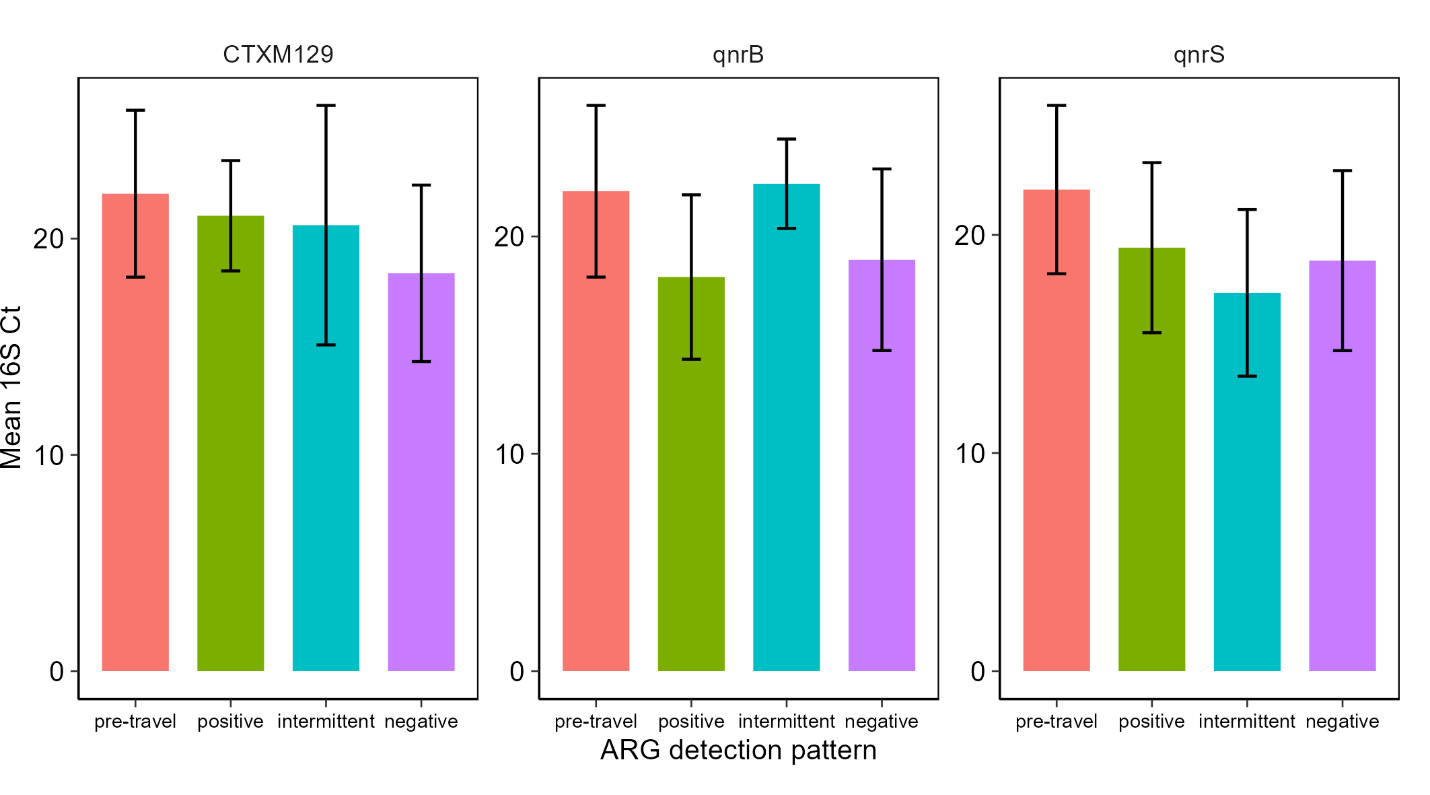
**

**Supplementary Fig. 14 Mean 16S rRNA gene Ct values across ARG detection patterns.** Bar plots display the mean 16S rRNA gene Ct for samples grouped by detection pattern (pre-travel; positive: AMR gene–positive; intermittent: intermittently negative; negative: AMR gene–negative) and stratified by gene (qnrS = *qnrS*, qnrB = *qnrB*, and CTXM129 = *bla*_CTX-M_ group 1, 2 and 9). For each gene and pattern category, 16s Ct values were averaged across all samples assigned to that category. Error bars indicate the standard deviation.


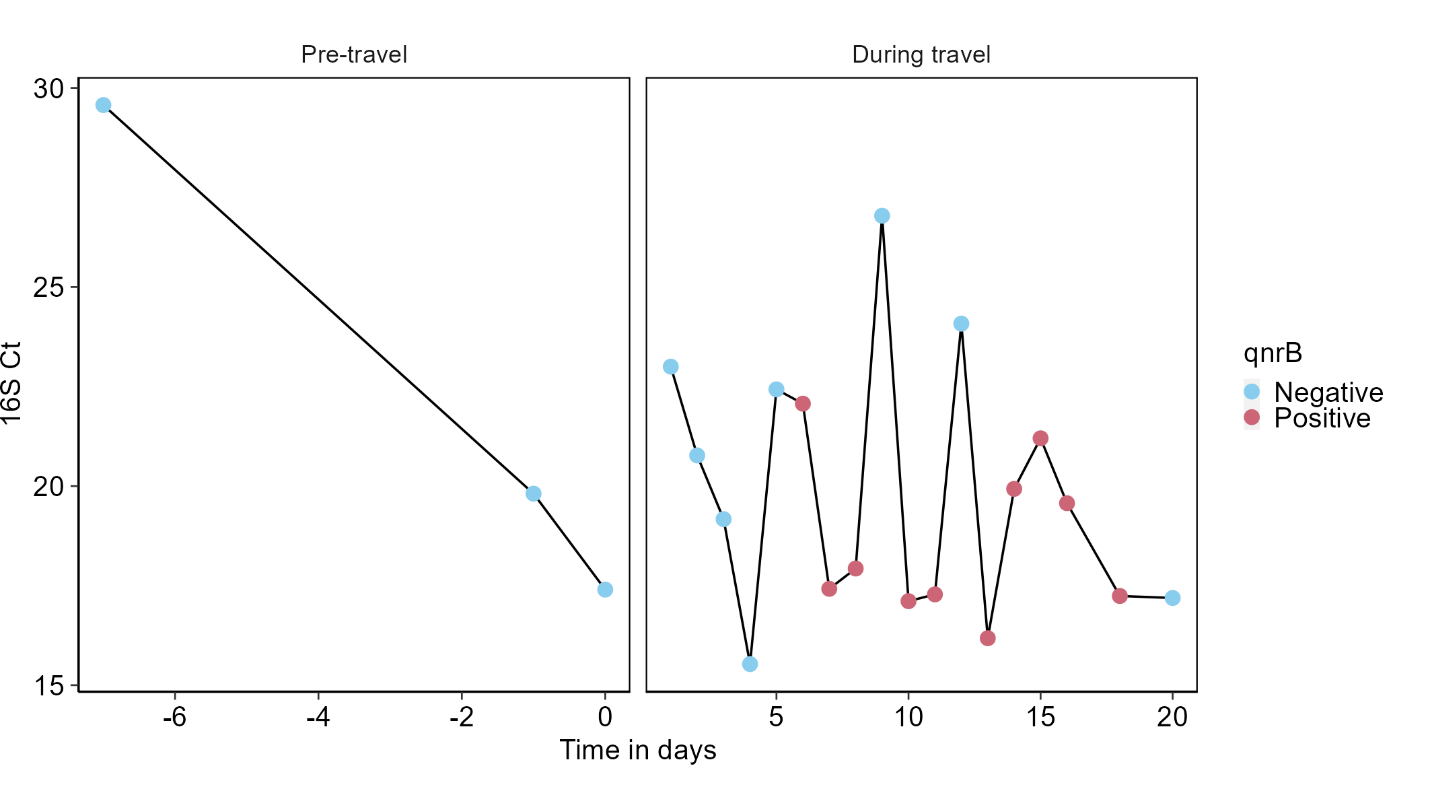


**Supplementary Fig. 15 Longitudinal 16S rRNA gene Ct dynamics in an individual traveller.** Line and point plot showing 16S rRNA gene Ct values across sequential time points for participant Tr03. DNA load of the samples was determined by the PCR Ct value obtained for 16S rDNA. A higher Ct value represents a lower DNA load. Points are coloured by *qnrB* detection status, and lines connect samples over time. Data are faceted by travel period (T0 = Pre-travel, T1 = During travel)

**
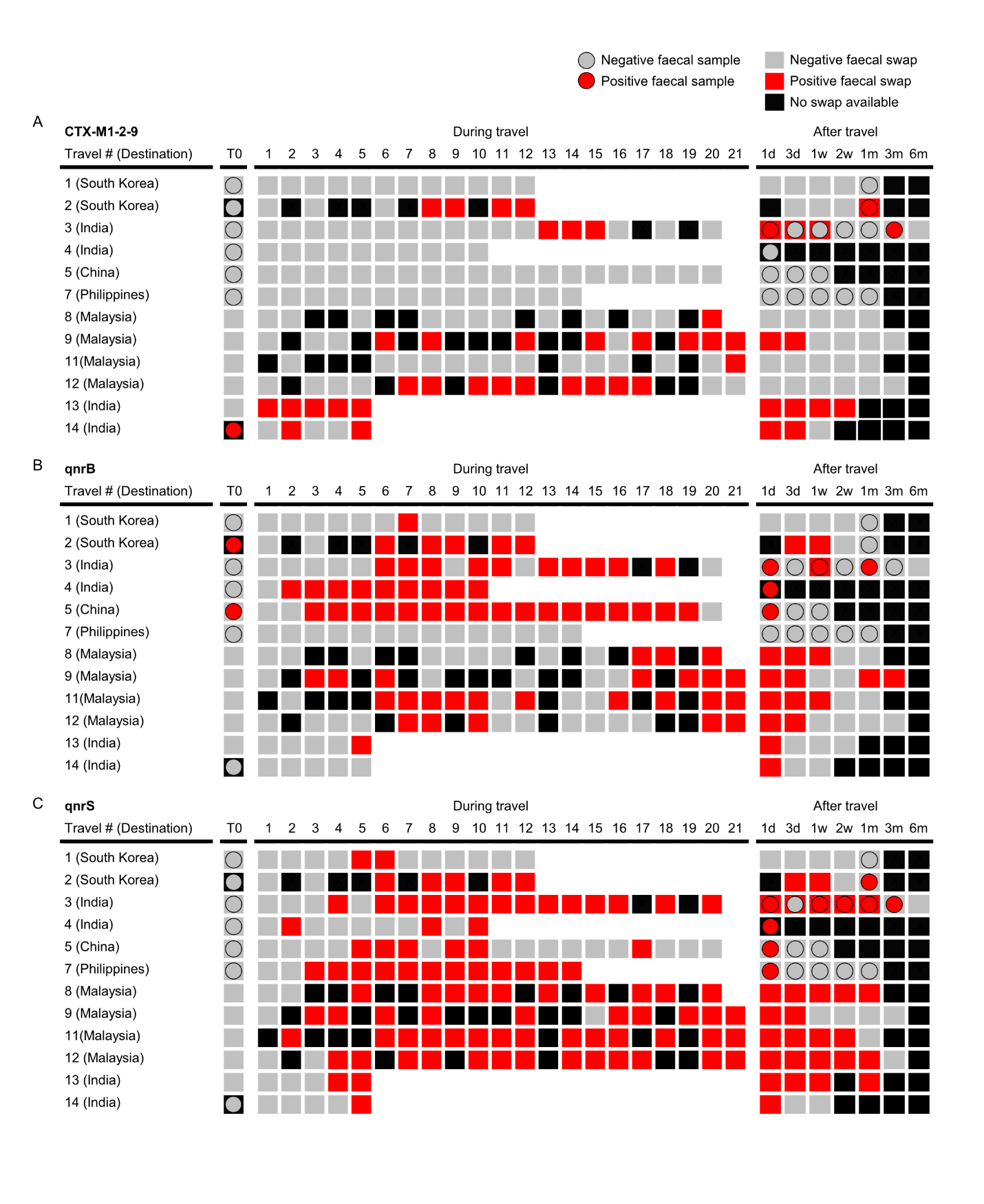
**

**Supplementary Fig. 16** **Presence of (A) *bla*_CTX-M_, (B) *qnrB* and *qnrS* (C) genes in faecal swabs and samples before, during or after international travel.** Squares represent faecal swabs and circles represent faecal samples. Grey colour indicates negative detection, and red colour indicates positive detection. Black squares denote time points at which no faecal swab was collected. T0: before travel; 1d: 1 day; 3d: 3 days; 1w: 1 week; 2w: 2 weeks; 1m: 1 month; 3m; 3 months; 6m: 6 months.

**
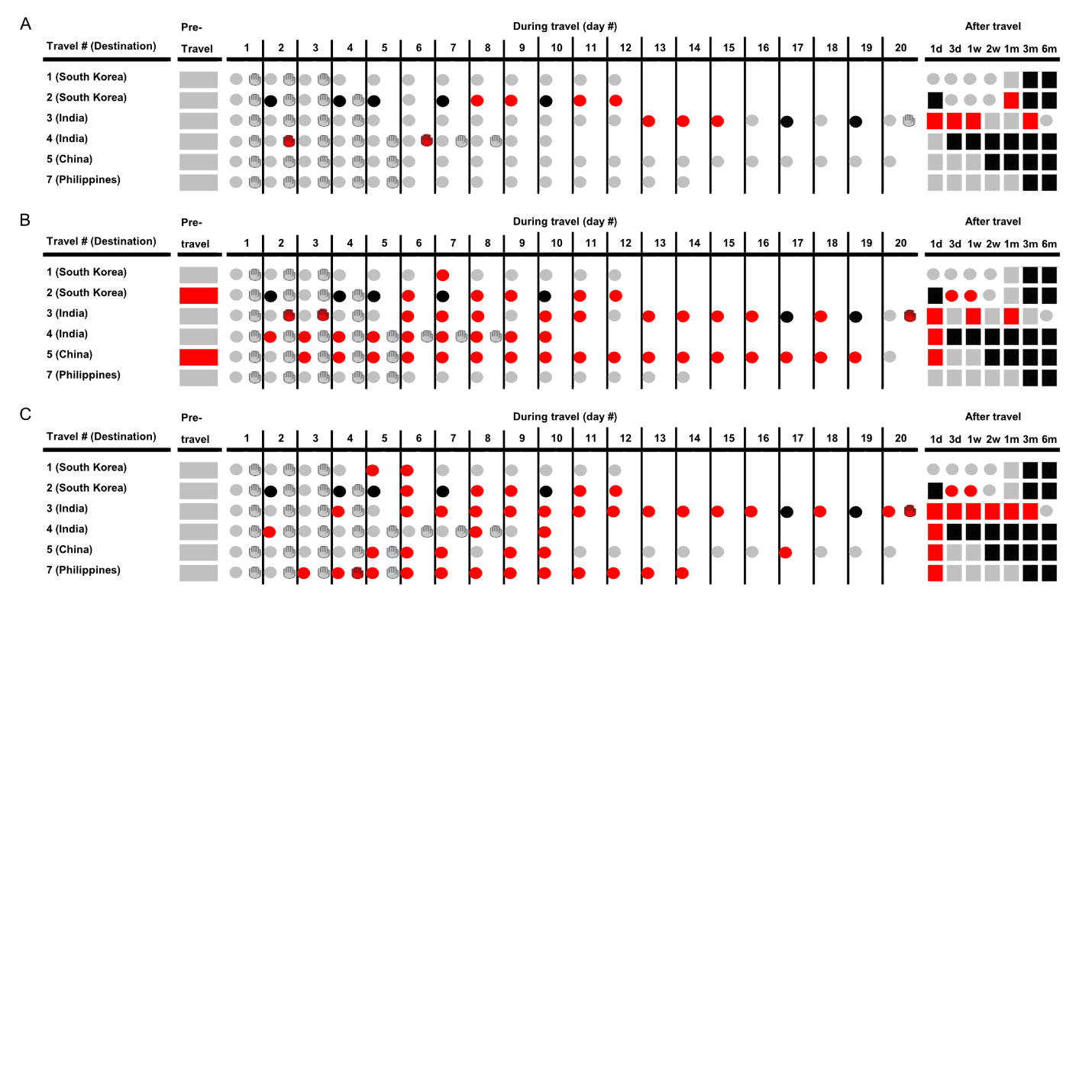
Supplementary Fig. 17 Presence of (A) *bla*_CTX-M_, (B) *qnrB* and (C) *qnrS* genes in faecal swab/sample or hand skin swab metagenomes before, during or after international travel.** Square, circle and hand symbols represent faecal samples, faecal swabs, and hand skin swabs respectively. Grey symbols represent samples which were negative, while red symbols represent those which were positive. Black symbols represent time points at which a faecal sample or swab was not collected. Only hand skin swabs which were collected are shown in the figure. 1d: 1 day; 3d: 3 days; 1w: 1 week; 2w: 2 weeks; 1m: 1 month; 3m; 3 months; 6m: 6 months.

**
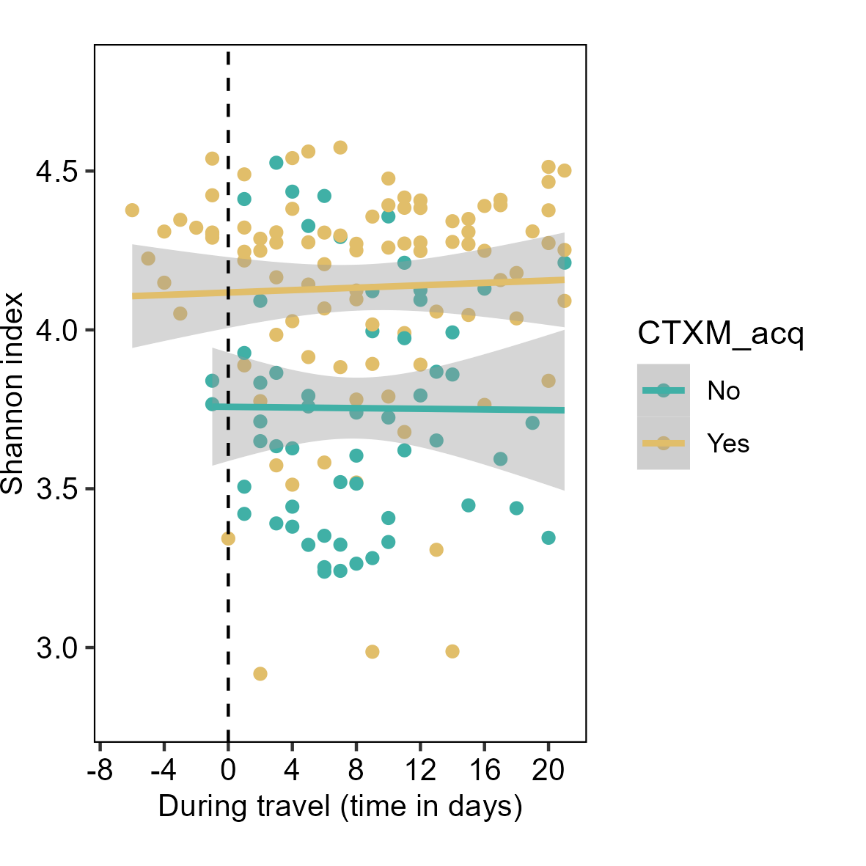
**

**Supplementary Fig. 18 Comparison between samples of travellers that did or did not acquire *bla*_CTX-M_ during their travels.** Dots represent individual samples and are coloured yellow for all samples of travellers that acquired *bla*_CTX-M_ at any given time-point during travel or green for all samples of travellers that remained negative for *bla*_CTX-M_. The standard error (SE) confidence band is displayed in grey.

**
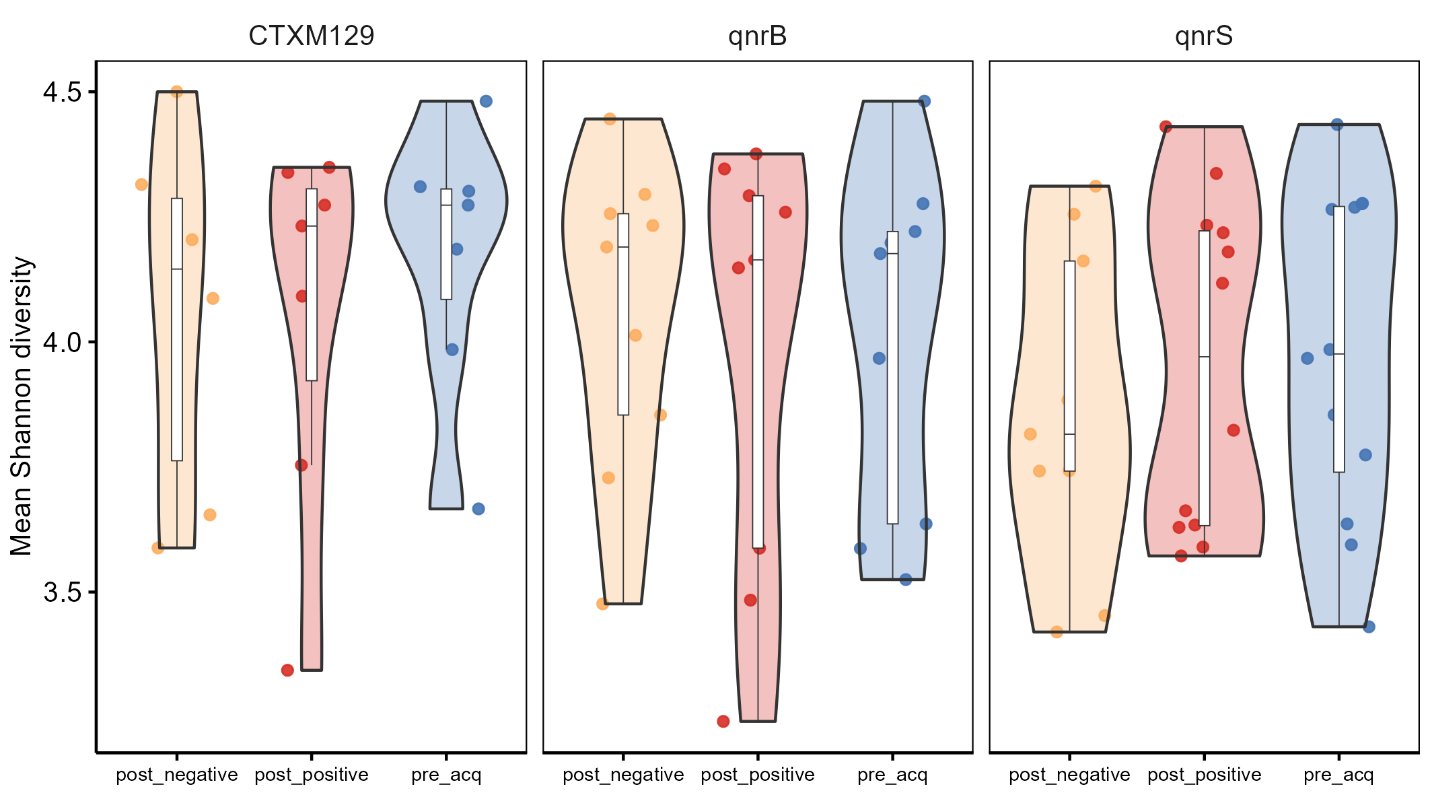
**

**Supplementary Fig. 19 Microbial diversity dynamics associated with ARG acquisition and persistence.** Shannon diversity is shown across acquisition phases (pre-acq: phase before AMR gene acquisition; post-positive: post-acquisition phase positive for the AMR gene; post-negative: post-acquisition phase negative for the AMR gene), faceted by gene. Points represent individual samples, violins depict the distribution of values. Box plots denote the median, IQR, and 95% quantiles.


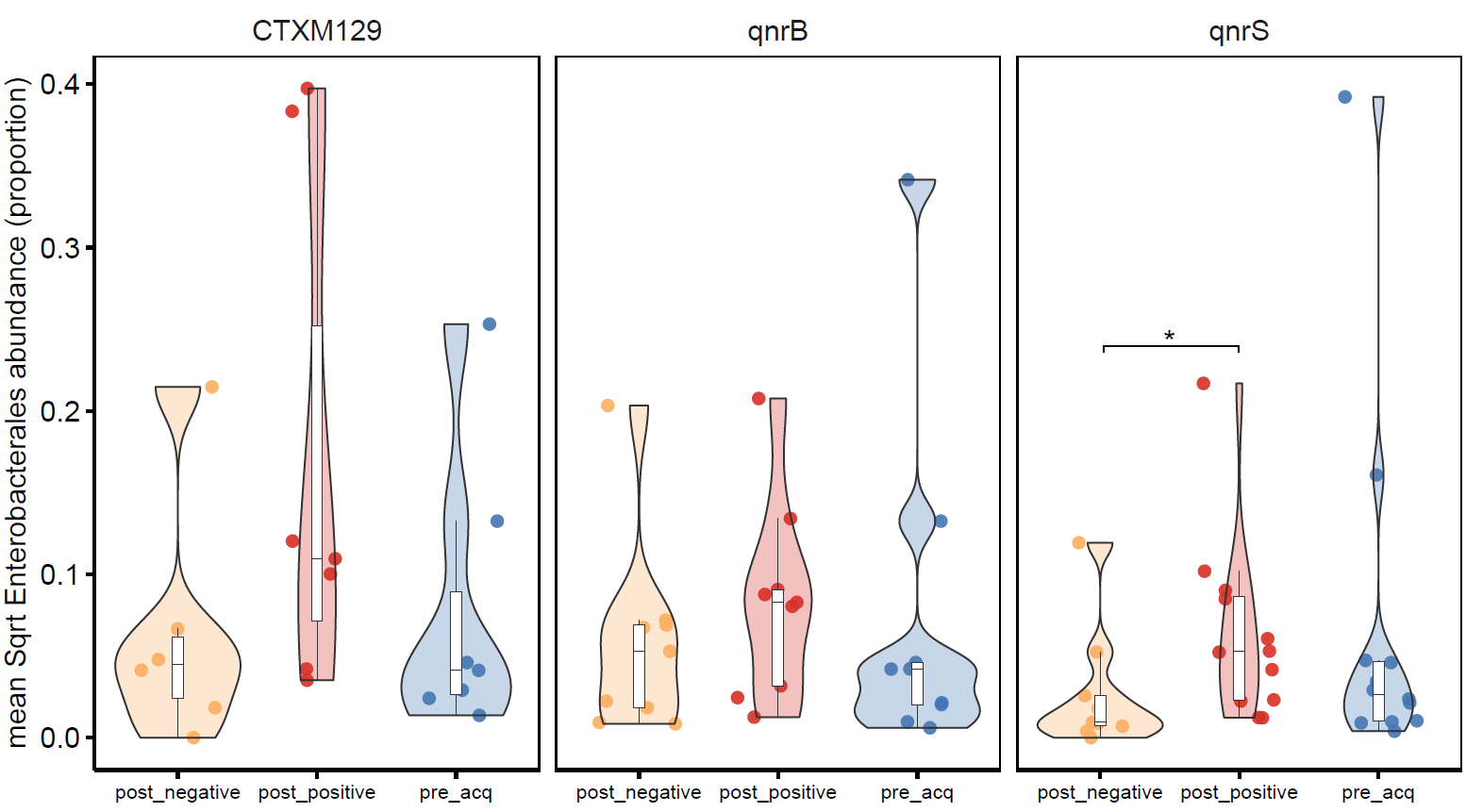
**Supplementary Fig. 20 Enterobacterales abundance across ARG acquisition phases.** Square-root–transformed mean Enterobacterales relative abundance is shown across acquisition phases (pre-acq: phase before AMR gene acquisition; post-positive: post-acquisition phase positive for the AMR gene; post-negative: post-acquisition phase negative for the AMR gene), faceted by gene. Points represent individual samples, violins show distributional structure. Box plots denote the median, IQR, and 95% quantiles. Statistical comparisons were performed using paired, two-sided Wilcoxon signed-rank tests with Benjamini–Hochberg correction (* adjusted P < 0.05)**
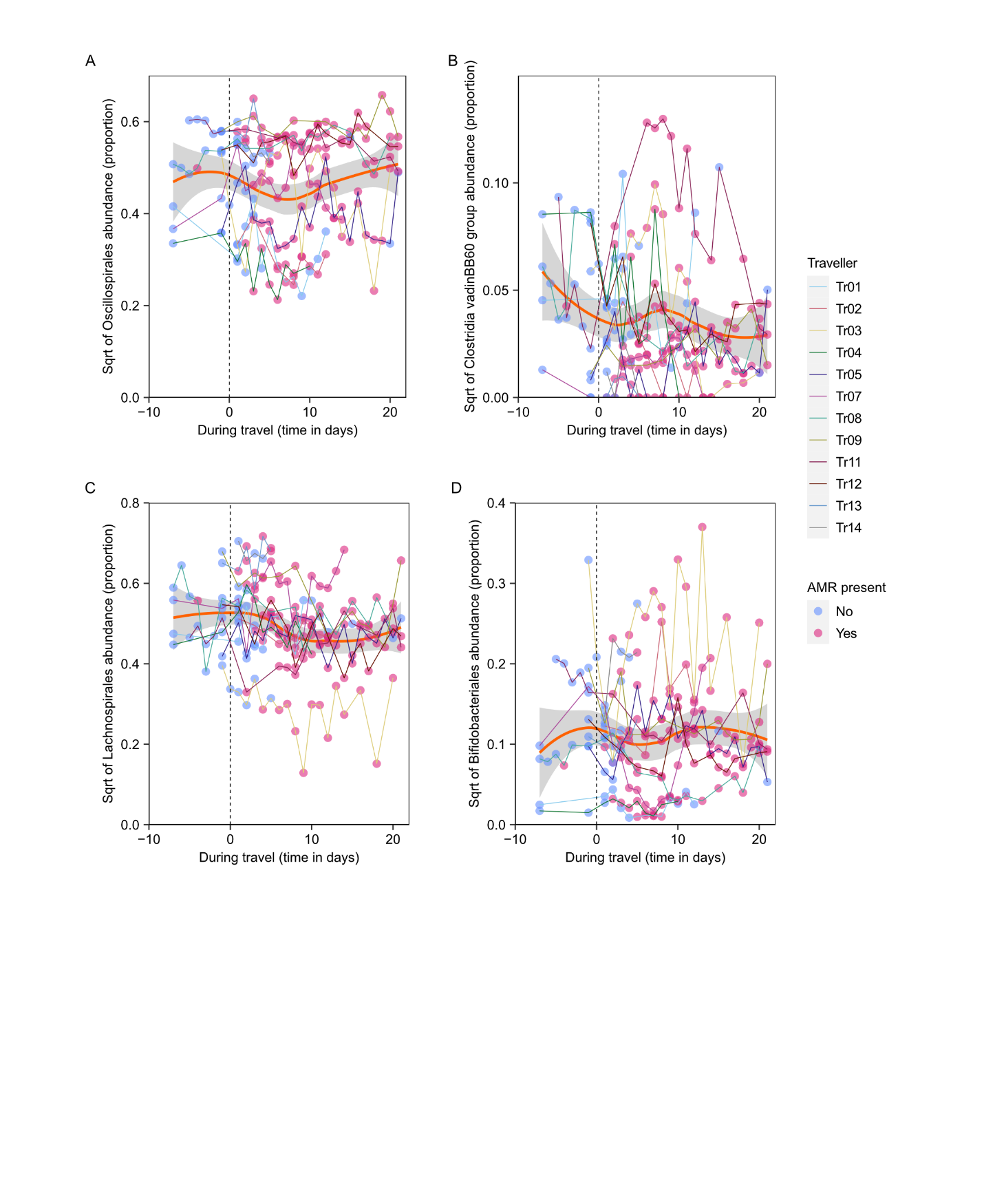
Supplementary Fig. 21 Relative abundance profiles** of *Oscillospirales* (A), Clostridia vadinBB60 group (B), *Lachnospirales* (C) and *Bifidobacteriales* (D) over time, from pre-travel (day -7 to day 0) till during travel (day 1 to day 21). Orange line depicts fitted Locally Estimated Scatterplot Smoothing (LOESS) curve. Dots connecting the lines depict the individual samples and are coloured pink when at least one of the studied ARGs was detected and blue when the sample was negative for all ARGs. Sqrt = Square root. The standard error (SE) confidence band is displayed in grey.
